# Genome-Wide Transcription Start Site Mapping and Promoter Assignments to a Sigma Factor in the Human Enteropathogen *Clostridioides difficile*

**DOI:** 10.3389/fmicb.2020.01939

**Published:** 2020-08-13

**Authors:** Olga Soutourina, Thomas Dubois, Marc Monot, Pavel V. Shelyakin, Laure Saujet, Pierre Boudry, Mikhail S. Gelfand, Bruno Dupuy, Isabelle Martin-Verstraete

**Affiliations:** ^1^Laboratoire Pathogenèses des Bactéries Anaérobies, Institut Pasteur, UMR CNRS 2001, Université de Paris, Paris, France; ^2^Institut Universitaire de France, Paris, France; ^3^Université Paris-Saclay, CEA, CNRS, Institute for Integrative Biology of the Cell (I2BC), Gif-sur-Yvette, France; ^4^Institute for Information Transmission Problems, Moscow, Russia; ^5^Skolkovo Institute of Science and Technology, Moscow, Russia

**Keywords:** transcription initiation, transcription unit architecture, sigma factors, sigma 54, amino acid catabolism

## Abstract

The emerging human enteropathogen *Clostridioides difficile* is the main cause of diarrhea associated with antibiotherapy. Regulatory pathways underlying the adaptive responses remain understudied and the global view of *C. difficile* promoter structure is still missing. In the genome of *C. difficile* 630, 22 genes encoding sigma factors are present suggesting a complex pattern of transcription in this bacterium. We present here the first transcriptional map of the *C. difficile* genome resulting from the identification of transcriptional start sites (TSS), promoter motifs and operon structures. By 5′-end RNA-seq approach, we mapped more than 1000 TSS upstream of genes. In addition to these primary TSS, this analysis revealed complex structure of transcriptional units such as alternative and internal promoters, potential RNA processing events and 5′ untranslated regions. By following an *in silico* iterative strategy that used as an input previously published consensus sequences and transcriptomic analysis, we identified candidate promoters upstream of most of protein-coding and non-coding RNAs genes. This strategy also led to refine consensus sequences of promoters recognized by major sigma factors of *C. difficile*. Detailed analysis focuses on the transcription in the pathogenicity locus and regulatory genes, as well as regulons of transition phase and sporulation sigma factors as important components of *C. difficile* regulatory network governing toxin gene expression and spore formation. Among the still uncharacterized regulons of the major sigma factors of *C. difficile*, we defined the SigL regulon by combining transcriptome and *in silico* analyses. We showed that the SigL regulon is largely involved in amino-acid degradation, a metabolism crucial for *C. difficile* gut colonization. Finally, we combined our TSS mapping, *in silico* identification of promoters and RNA-seq data to improve gene annotation and to suggest operon organization in *C. difficile*. These data will considerably improve our knowledge of global regulatory circuits controlling gene expression in *C. difficile* and will serve as a useful rich resource for scientific community both for the detailed analysis of specific genes and systems biology studies.

## Introduction

*Clostridioides difficile* (formerly *Clostridium difficile*) is an emerging human enteropathogen causing nosocomial antibiotic-associated diarrhea in adults (Carroll and Bartlett, 2011). This pathogen became a key public health issue worldwide. Alarming incidence of *C. difficile* infections was further accentuated by the recent emergence of antibiotic resistance, of hypervirulent epidemic strains broadening the population at risk and severity of disease, high rate of recurrent infection as well as an overall aging of population in industrial countries (Rupnik et al., 2009; Banawas, 2018). This anaerobic, spore-forming, Gram-positive bacterium can be found in soil and aquatic environments as well as in intestinal tracts of humans and animals (Keessen et al., 2011). In humans, the *C. difficile* carriage may be asymptomatic, but when symptoms appear, they can vary from mild diarrhea to life-threatening pseudomembranous colitis. The main *C. difficile* virulence factors are two toxins, TcdA and TcdB, produced by toxigenic strains (Vedantam et al., 2012). A binary toxin CDT is also present in some isolates, as well as additional factors that help colonization, like adhesins, pili, and flagella (Janoir, 2016). Despite recent efforts of research community, a lot of questions remains unanswered on the regulatory processes responsible for the adaptation of *C. difficile* inside the host. This information is largely missing and urgently needed for our understanding of the success of this pathogen and development of new diagnostic and therapeutic strategies.

Recent advances in high throughput approaches resulted in the accumulation of new invaluable data on the regulatory strategies of pathogenic bacteria at genomic, transcriptomic and metabolomics levels (Lo et al., 2017; Saliba et al., 2017; Hor et al., 2018). RNA-seq studies notably revealed high complexity of the transcriptome landscape in bacteria (Sorek and Cossart, 2010; Saliba et al., 2017). The precise exploration and interpretation of this large amount of data is essential to improve our understanding of the *C. difficile* infection cycle. One of the first steps towards the establishment of *C. difficile* regulatory pathways is the definition of the transcriptional map at a genome-wide level. In particular, the analysis of transcriptome data combined with *in silico* predictions could provide key information on molecular details of regulatory mechanisms including promoter sequences, type of sigma factor associated to the RNA polymerase (RNAP) involved in the initiation of transcription, as well as other regulatory elements. The genome-wide transcriptional start site (TSS) mapping allows the determination of transcriptional start positions at single-nucleotide resolution (Wurtzel et al., 2010; Sharma and Vogel, 2014). Two methods developed in 2010 have proven their validity for TSS mapping. Differential RNA-seq (dRNA-seq) method largely used in prokaryotes includes two enzymatic steps, i.e., terminator 5′-phosphate-dependent exonuclease (TEX) followed by tobacco acid pyrophosphatase (TAP) treatment for adapter ligation, while 5′-end RNA-seq compares two samples treated or not with TAP enzyme to distinguish between primary 5′-triphosphate and processed 5′-monophosphate transcripts (Sharma et al., 2010; Jager et al., 2014; Sharma and Vogel, 2014; Papenfort et al., 2015; Babski et al., 2016). The 5′-end RNA-seq approach (Wurtzel et al., 2010) that we are using in this study has been successfully implemented for the TSS mapping in Achaea and bacteria including pathogenic and non-pathogenic Listeria (Wurtzel et al., 2012a), *Pseudomonas aeruginosa* (Wurtzel et al., 2012b), *Streptococcus agalactiae* (Rosinski-Chupin et al., 2015), *Streptococcus pyogenes* (Rosinski-Chupin et al., 2019). The detection of TSS implies the presence of a promoter in its upstream region. This promoter element will define the site directing the RNAP for the initiation of transcription and represents a key element to understand the regulation of gene expression in bacteria. Promoters differ at their consensus sequences depending on the interchangeable RNAP sigma factor, which provides DNA recognition specificity (Burgess and Anthony, 2001).

During its infection cycle, *C. difficile* have to face changing conditions including variations in pH, oxygen content, osmolarity and exposure to various antimicrobial compounds (Abt et al., 2016). The *C. difficile* 630 genome carries 14 genes encoding sigma factors including two copies of housekeeping SigA and several alternative sigma factors allowing the initiation of transcription under various physiological conditions (Sebaihia et al., 2006) (Table 1). These sigma factors belong to the sigma 70 family except for one of them, SigL, belonging to the sigma 54 family (Gruber and Gross, 2003). Four specific sigma factors of sporulation, SigF and SigG in the forespore and SigE and SigK in the mother cell and the sigma factor of transition phase, SigH, which is involved in the initiation of sporulation are present in *C. difficile* and their inactivation leads to an asporogenous phenotype (Saujet et al., 2011, 2013; Fimlaid et al., 2013). The sigma factor of the general stress response, SigB, is involved in adaptive strategy during gut colonization and plays a role in resistance to low pH, to antimicrobial peptides, to reactive oxygen species and nitric oxide as well as in oxygen tolerance (Kint et al., 2017). TcdR is an alternative sigma factor required for toxin genes expression (*tcdA* and *tcdB*) while *tcdR* itself is transcribed by RNAP with SigD, which controls flagellar synthesis and motility (Mani and Dupuy, 2001; El Meouche et al., 2013). 3 extracytoplasmic function (ECF) sigma factors, SigV, CsfU and CsfT are also present (Ho and Ellermeier, 2011; Sineva et al., 2017). The regulons of several RNAP sigma factors have been recently defined in *C. difficile* including sporulation specific sigma factors, the general stress-response sigma factor, SigB and the motility-associated sigma factor, SigD (Saujet et al., 2011; El Meouche et al., 2013; Pereira et al., 2013; Saujet et al., 2013; Kint et al., 2017). Only two studies have previously shown a role of SigL, belonging to the Sigma 54 family, in the degradation of cysteine associated with a control of toxin production (Dubois et al., 2016; Gu et al., 2017; Nie et al., 2019). Interestingly, the genes for 24 transcriptional activators (named EBP for enhancer binding proteins activating SigL-dependent promoters) containing a AAA^+^ domain, which is responsible for ATP hydrolysis and their interaction with SigL (Francke et al., 2011), are present in the genome of *C. difficile*. Only two EBPs, CsdR and PrdR, controlling cysteine and proline catabolism, respectively, have been experimentally characterized in *C. difficile* (Bouillaut et al., 2013; Gu et al., 2017; Gu et al., 2018).

**TABLE 1:** Sigma factors encoded in the genome of *C. difficile* strain 630.

Gene ID	Gene name	Function	Group*	% identity/similarity with sigma factors of *B. subtilis*	References
CD1455	*sigA1*	SigA, exponential growth	1	65/77	Saujet et al., 2011
CD1498	*sigA2*	SigA, stationary phase	1	56/74	Saujet et al., 2011
CD0011	*sigB*	SigB, general stress response	2	34/54	Kint et al., 2017
CD0266	*sigD*	SigD, flagella formation	3	30/53	El Meouche et al., 2013
CD3176	*sigL*	SigL, sigma 54 factor	NA	29/50	Dubois et al., 2016
CD0057	*sigH*	SigH factor of transition phase	3	63/78	Saujet et al., 2011
CD2643	*sigE*	SigE factor of sporulation	3	64/77	Pereira et al., 2013; Saujet et al., 2013
CD0772	*sigF*	SigF factor of sporulation	3	49/72	Pereira et al., 2013; Saujet et al., 2013
CD2642	*sigG*	SigG factor of sporulation	3	68/86	Pereira et al., 2013; Saujet et al., 2013
CD1230	*sigK*	SigK factor of sporulation	3	56/77	Pereira et al., 2013; Saujet et al., 2013
CD1558	*csfV/sigV*	SigV, ECF sigma factor, resistance to lyzozyme	4	57/76	Ho and Ellermeier, 2011
CD1887	*csfU*	ECF sigma factor SigW-like	4	30/48	Ho and Ellermeier, 2011
CD0677	*csfT*	ECF sigma factor	4	–	Ho and Ellermeier, 2011
CD0659	*tcdR*	Sigma factor of toxin genes	5	–	Mani and Dupuy, 2001

*Eight additional Sigma factors are annotated in the genome of *C. difficile* strain 630 (Sebaihia et al., 2006). The genes *CD0359, CD0435, CD0510, CD1094, CD1878, CD2223, CD3329*, and *CD3371* encoding these sigma factors are associated with conjugative transposon. Group^∗^ of σ^70^ family sigma factors according to the classification by Gruber and Gross (2003). “NA” not applicable.*

By combining *in silico* analysis, RNA-seq and genome-wide promoter mapping, we have recently identified more than 200 non-coding RNAs (ncRNAs) in *C. difficile* from different functional classes including riboswitches, *trans*- and *cis*-acting antisense RNAs (Soutourina et al., 2013). However, the global view of *C. difficile* promoter structure is still missing. To fill the gap in our current understanding of *C. difficile* genes and transcriptional unit structure and regulation, we present here a transcriptional map of the *C. difficile* genome including the identification of TSSs, operon structures and promoter motifs. We also defined the SigL regulon. These data would considerably improve our knowledge on the regulons of the major sigma factors and on the global regulatory circuits that control gene expression in *C. difficile*. This work will be essential for genome-wide and gene-specific studies of the regulatory mechanisms in this emerging pathogen.

## Materials and Methods

### Bacterial Strains and Growth Conditions

*Clostridioides difficile* strains were grown anaerobically (5% H_2_, 5% CO_2_, and 90% N_2_) in TY (Bacto tryptone 30 g.l^–1^, yeast extract 20 g.l^–1^, pH 7.4) or Brain Heart Infusion (BHI, Difco). For solid media, agar was added to a final concentration of 17 g.L^–1^. When necessary, cefoxitin (Cfx; 25 μg/ml), erythromycin (Erm; 2.5 μg/ml) and thiamphenicol (Tm; 15 μg/ml) were added to *C. difficile* cultures. Strains and plasmids used in this study are listed in Supplementary Table S1.

### Volatile Fatty Acid Analysis

The end products of fermentation were detected by gas-liquid chromatography. Strain 630Δ*erm* and the *sigL::erm* mutant were grown in TY for 48 h at 37°C. After centrifugation, supernatants were recovered and mixed with sulfuric acid and ether to extract the volatile fatty acids. For each sample, 5 μl of the supernatants was injected in a gas chromatograph (CP-3380; Varian) equipped with a flame ionization detector and connected to an integrator (model C-55A; Shimadzu). A glass column (2 m by 4 mm) packed with 10% SP 1000 plus 1% H_3_PO_4_ on Chromosorb W AW (100/120 mesh) was used. The instrument was operated at 170°C for 18 min. The operating conditions were as follows: injector temperature, 200°C; detector temperature, 200°C; carrier gas (nitrogen); flow rate, 30 ml min^–1^. Each peak of the GLC patterns was identified by the retention time compared with that obtained with standard (2-Methylpentanoic acid at 10 mM). The amounts of fatty acids were calculated by comparison with an internal standard (Carlier and Sellier, 1989).

### RNA Extraction, Quantitative Real-Time PCR and 5′ RACE

For the 5′-end RNA-seq experiment, total RNA was isolated from *C. difficile* 630Δ*erm* strain grown in TY medium either after 4 h or 10 h of growth or under starvation conditions that correspond to a 1 h resuspension of exponentially grown cells (6 h of growth) into PBS buffer for 1 h at 37°C as previously described (Andre et al., 2008; Soutourina et al., 2013). 12 ml of culture for each condition have been used yielding at least 100 μg of total RNA. The analysis of genes controlled by SigL was performed with RNA extracted from cells of strain 630Δ*erm* or 630Δ*erm sigL*::*erm* mutant (Dubois et al., 2016) after 4 h of growth in TY. After centrifugation, the culture pellets were resuspended in RNApro^TM^ solution (MP Biomedicals) and RNA extracted using the FastRNA Pro Blue Kit, according to the manufacturer’s instructions. The RNA quality was determined using RNA 6000 Nano Reagents (Agilent). Quantitative real-time PCR analysis was performed as previously described (Saujet et al., 2011). In each sample, the quantity of cDNAs of a gene was normalized to the quantity of cDNAs of the *dnaF* gene (*CD1305*) encoding DNA polymerase III. The relative change in gene expression was recorded as the ratio of normalized target concentrations (ΔΔCt) (Livak and Schmittgen, 2001). 5′ RACE experiments were performed as previously described (Soutourina et al., 2013).

### RNA-Seq and 5′-End RNA-Seq

**FIGURE 1:**
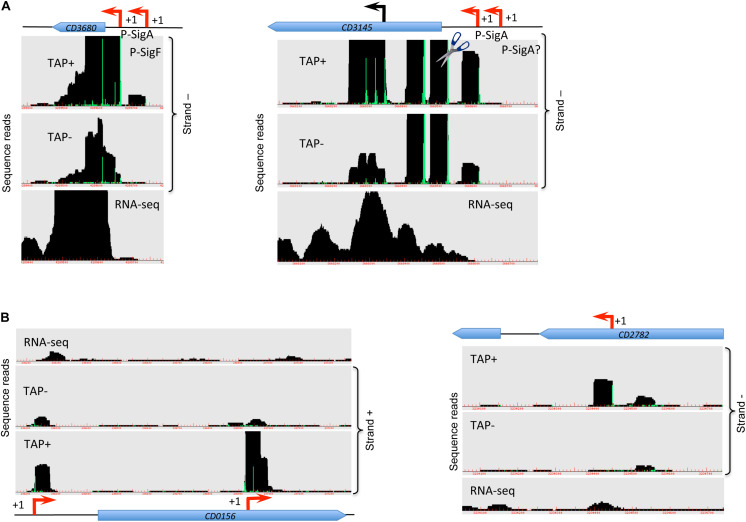
Visualization of TSS mapping of dual **(A)** and internal **(B)** promoters. Representative examples of 5′-end RNA-seq (TAP−/TAP+ profile comparison) and RNA-seq data for dual tandem TSS and internal TSS inside the coding sequences are shown in panel **(A)** and **(B)**, respectively. Cov2HTML (Monot et al., 2014) was used for the visualization. On a RNA-seq and 5′-end RNA-seq sequence read mapping visualization, coding sequences are indicated by blue arrows. The 5′-end RNA-seq data for either positive “strand +” or negative “strand −” strands are presented in the panels. The TSS identified by 5′-end RNA-seq are indicated by red broken arrows and potential processing sites are indicated by scissors mark. Sigma factor consensus associated with a given TSS is indicated. The TSS corresponds to a position with significantly greater number of reads in TAP+ sample, potential cleavage site corresponds to position with large number of reads in both TAP− and TAP+ samples. 5′-end RNA-seq data show 51-bp reads matching to the 5′-transcript ends, while RNA-seq data show reads covering whole transcript.

**FIGURE 2:**
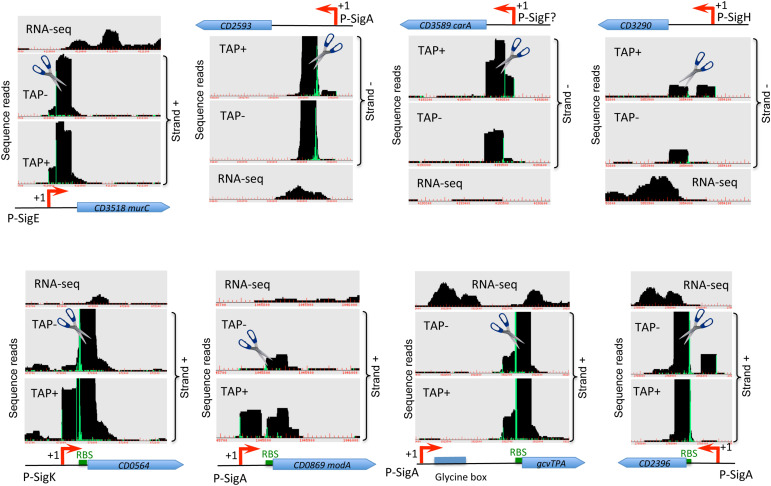
Examples of cleavage sites detected by TSS-mapping. Representative examples of 5′-end RNA-seq (TAP-/TAP+ profile comparison) and RNA-seq data for potential processing profiles are shown. The RNA-seq and 5′-end RNA-seq data visualization is presented as in Figure 1. Potential cleavage site shown by scissors mark corresponds to a position with large number of reads in both TAP– and TAP+ samples. RBS are shown by green boxes to highlight the positions of potential cleavage sites in the proximity or inside RBS.

**FIGURE 3:**
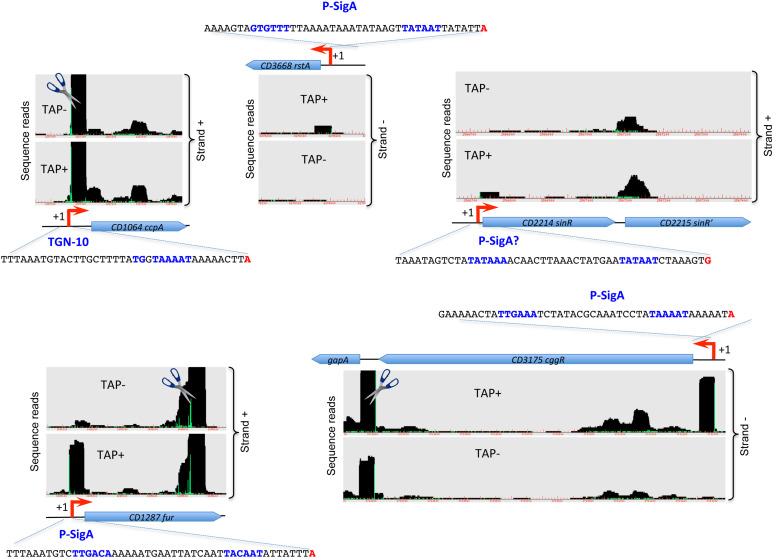
Examples of promoters controlling regulatory genes detected by TSS-mapping. Representative examples of 5′-end RNA-seq (TAP–/TAP+ profile comparison) data for the identification of TSS for genes encoding important transcriptional regulators are shown. The 5′-end RNA-seq data visualization is presented as in Figure 1. The sequence of promoter region is shown upstream of TSS with the –35 and –10 promoter elements indicated in blue and TSS indicated in red.

Non-orientated library for whole transcript analysis by RNA-seq was realized on a RNA sample extracted from *C. difficile* 630Δ*erm* strain grown at the late-exponential phase (6 h of growth) as previously described (Wurtzel et al., 2010). For 5′-end RNA-seq, two strand-specific cDNA libraries were generated from mixed RNA samples depleted for rRNAs and treated or not with Tobacco Acid Pyrophosphatase (TAP +/−), as previously described (Soutourina et al., 2013). TAP converts 5′-triphosphates into 5′-monophosphates allowing sequencing adaptor ligation and thus the enrichment with primary transcript reads in the library treated with TAP. The TAP+/− library construction for high-throughput sequencing (5′-end RNA-seq) was realized on mixed sample combining RNAs extracted from three different growth conditions including exponential growth (4 h), entry to a stationary phase (10 h) and nutriment starvation (1h incubation in PBS buffer). We prioritized depth of coverage by combining several conditions in a single 5′-end RNA-seq to maximize the number of genes expressed and thus the number of detectable TSS. 15 μg of total RNA treated with TurboDNAse (Ambion) was used for depletion of ribosomal RNA with the MicrobExpress kit (Ambion) following the manufacturer instructions. RNA depleted for rRNA was divided into two similar fractions and 1500 ng of this partially purified RNA was used for each library preparation. To convert the 5′PPP ends in 5′P ends, RNA was denatured 10 min at 65°C, placed on ice and incubated 1 h at 37°C with 10 units of TAP (Epicenter) (TAP+ library). For TAP− library construction, RNA depleted for rRNA was incubated with buffer alone under the same conditions. Products were purified by phenol/chloroform extraction and ethanol precipitation. cDNA library construction for Illumina sequencing was performed as previously described (Sahr et al., 2012). The Illumina reads were first scanned using Tagdust for adaptor removal. To eliminate reads matching the rDNA, sequence reads were mapped to the rDNA operon sequences of *C. difficile* 630 strain using the Bowtie software. Remaining sequencing reads were mapped to the *C. difficile* genome as previously described (Soutourina et al., 2013). We have set up a semi-automatic procedure for data analysis. The data were visualized using COV2HTML (Monot et al., 2014) at a strand-specific manner (for 5′-end RNA-seq libraries) or as a whole transcript coverage map (for RNA-seq): http://mmonot.eu/COV2HTML/visualisation.php?str_id=-44. This interface allows to visualize the accumulation of the first bases of the reads, which is the signal generated by the 5′-end RNA-seq approach. The sequences were localized on the genome and formed peaks (shown in green, see Figures 1–3, 6) that were easily detectable because the background noise was low. All transcriptional start signals detected by 5′-end RNA-seq were inspected manually to identify potential TSS and cleavage sites helped with a score that is the first base coverage ratio at the given position between the two conditions TAP+/TAP− (normalized by the number of total reads). The analysis consisted of scanning the genome systematically and each time a green peak was detected, we zoomed in the region to access the surrounding sequences for detection of promoter motifs. Following criteria have been taken into consideration for the TSS validation: TAP+/TAP− ratio (cut-off value of 1.5 in 90% of cases), the identification of promoter motifs at defined distance upstream of TSS (*in silico* promoter prediction score of at least 2 with −10 and −35 boxes positions from TSS separated by 16–18 bp or −12 and −24 consensus motif positions for the SigL promoters), the TSS location with respect to the annotated gene (upstream of the RBS for protein-coding genes) and whole transcript RNA-seq data coverage. We expected a distribution of RNA-seq coverage signal from TSS position to the 3′-end of the transcript provided that the gene was expressed at sufficient level under tested conditions. In case of previously studied sigma factors, an additional criterion has been included on the differential expression in comparative transcriptome analysis between strain inactivated for a given sigma factor and wild type strain [SigH (Saujet et al., 2011), SigE-F-G-K (Saujet et al., 2013), SigD (El Meouche et al., 2013), SigB (Kint et al., 2017), Sigma 54 (present study)].

Then, we also used the PhageTerm software (Garneau et al., 2017) to automatically scan for significant peaks through a statistic module included (Supplementary Table S2). PhageTerm has been developed to statistically detect sequencing bias due to the way phages are packaged. One of these packaging mode (Pac) creates an accumulation of the first bases of sequences identical to the TSS signal. A transcript can be thought of as a small linear genome like a phage and then processed by PhageTerm. First, each gene locus is segmented according to coverage using a regression tree. Then, a gamma distribution is fitted to starting position coverage for each segment and an adjusted *p*-value is computed for each position. This two-parameter method is quite similar the one used in TSSAR, which uses Poisson distribution with a second parameter that depends on the local strength of expression (Amman et al., 2014). The analysis was limited to a region of 500 bases before each gene for process time constraints (160 computing days to scan 3000 genes). PhageTerm detected 856 TSS (Supplementary Figure S1), 74% (633) were already determined by semi-automatic procedure and 26% (233) were new. Of these 233, only 31 contained grounds for inclusion in the list according to TSS criteria described above. PhageTerm analysis results thus overlapped largely with the predictions of our semi-automatic analyses. Most of the remainder were potential cleavage sites. The complete experimental TSS raw data set was deposited in the SRA database with an accession number PRJNA626554.

### Microarray Design, DNA-Array Hybridization and Transcriptome Analysis

The microarray of *C. difficile* 630 genome was designed as previously described (Saujet et al., 2011) (GEO database accession number GPL10556). Transcriptome was performed using four different RNA preparations for each condition (630Δ*erm* and the *sigL::erm* mutant). Labeled DNA hybridization to microarrays and array scanning were done as previously described (Saujet et al., 2011). The complete experimental data set was deposited in the GEO database with a superseries record accession number GSE149245. All the slides were analyzed using R and limma software (Linear Model for Microarray Data) from Bioconductor project^1^. For each slide, we corrected background with the ‘normexp’ method, resulting in strictly positive values and reducing variability in the log ratios for genes with low levels of hybridization signal. Then, we normalized each slide with the ‘loess’ method (Smyth and Speed, 2003). To test for differential expression, we used the bayesian adjusted t-statistics and we performed a multiple testing correction of Benjamini and Hochberg based on the false discovery rate (FDR) (Benjamini and Hochberg, 1995). For the transcriptome data, a gene is considered as differentially expressed when the *p*-value is < 0.05.

### Strategy of Identification of Promoters Upstream of TSS

Identification of the consensus sequences for each of the sigma factors corresponding to transcription start sites (TSSs) was made using a modified procedure from Saujet et al. (2013). Positional weight matrices (PWMs) for the different sigma factors SigK, SigH, SigF, SigG, SigD, SigE and SigB were made based on a set of experimentally determined binding sites in *C. difficile* genome (Eckweiler et al., 2018) and for SigA and SigL based on *Bacillus subtilis* sites from DBTBS (Sierro et al., 2008). These PWMs, in addition to nucleotides in the proximal and distant promoter boxes, assign weights to distances between these boxes. Genome regions upstream of TSSs were scanned with these PWMs, the maximal allowed distance from a TSS to the proximal box was set to 15 nucleotides. For each sigma factor the highest scoring candidate site was retained, yielding a matrix of scores TSS × PWM. To account for the fact that PWMs have different distributions of scores, precluding direct comparison, all scores were transformed to Z-scores by subtracting the mean and dividing by the standard deviation of scores produced by the respective PWM on a set of random sequences. To account for uneven GC-content, for each upstream region, the set of random sequences was constructed by shuffling of its sequence 10000 times. In the case of sigma 54 (SigL), raw scores of sites that did not include known important dinucleotides GG and GC in the distal and proximal boxes, respectively (Nie et al., 2019), were artificially decreased by the maximal achievable score for this matrix for each mismatch. We considered independently two variants of PWMs for SigA, the standard two-box one and the extended one without the distal box, but with invariant TG preceding the proximal box. Finally, for each TSS we ranged sigma factors by their Z-scores and retained two sigma factors with maximal Z-scores as most probable regulators of the TSS.

To validate the assignment of sigma factors to promoters, we used the data on gene expression levels in mutants with knocked out sigma factors: SigH (Saujet et al., 2011), SigE-F-G-K (Saujet et al., 2013), SigD (El Meouche et al., 2013), SigB (Kint et al., 2017), Sigma 54 (present study). Quality of prediction was assessed using a confusion matrix. Rows in the matrix correspond to the actual sigma factors and columns to the *in silico* predicted sigma factors, so the correct predictions are located at the diagonal. A gene was assumed to be actually regulated by a sigma factor if its expression level changed by at least 10% in knockout mutants. For each TSS, candidate regulating sigma factors were predicted and ranged as described above. A value in a diagonal cell counts cases when the actual sigma factor was among two best ones predicted (or among three best ones if the two best corresponded to SigA and extended SigA motif). A value in an off-diagonal cell is the count of incorrect predictions with the column corresponding to the predicted sigma factor with the highest score, and the row corresponding to the actual sigma factor (or sigma factors if a gene changed its expression in several mutants).

## Results and Discussion

### Genome Wide Mapping of Transcriptional Start Sites by 5′-End RNA-Seq

A total of 3684 genes have been annotated in the genome of *C. difficile* strain 630 (Sebaihia et al., 2006; Monot et al., 2011). The main objective of this study was to provide accurate identification of transcriptional start sites (TSS) for a large number of *C. difficile* 630 transcriptional units. For genome-wide detection of TSS, we performed a differential 5′-end sequencing approach (5′-end RNA-seq). To increase the number of TSS identified, the 5′-end RNA-seq analysis was realized with mixed RNA samples extracted from *C. difficile* 630Δ*erm* cells harvested during exponential growth phase (4 h), at the onset of stationary phase (10 h) and under nutrient starvation conditions. We pooled together three samples from different growth conditions before sequencing to cover the *C. difficile* transcription map, a strategy largely used for similar studies (Rosinski-Chupin et al., 2015, 2019). The goal was to identify the TSS position and not to analyze the differential gene expression. The lack of replicates could be a problem for genes expressed exclusively in one of tested conditions, however, based on our previous transcriptome analysis and gene-specific studies, we assume that the great part of genes is expressed at least in two or three samples. This study is not exhaustive but provide valuable information about TSS positions for a part of *C. difficile* genes that is useful for further investigations of this pathogen.

This approach allowed us to identify using both the PhageTerm software and manual inspection of sequence data a total of 1562 potential TSS upstream of *C. difficile* strain 630 genes including 274 TSS upstream of non-coding RNA genes (Soutourina et al., 2013) (Supplementary Tables S2, S3). Among TSS associated with ncRNA genes, 66 are located upstream riboswitches, 7 upstream rRNA and 24 upstream tRNA genes (Supplementary Table S3). For the cluster of highly expressed tRNA genes, a number of suggested TSS could correspond to cleavage sites representing processing events during transcript maturation process.

Together with automatic *in silico* approaches (Amman et al., 2014; Jorjani and Zavolan, 2014), manual data curation still remains valuable for gene annotation and data interpretation and validation. For the TSS mapping data analysis we have used a semi-automatic procedure. From the 5-end RNA-seq data the accumulation of the first bases of the reads has been visualized as green peaks in our visualization interface. The genome has been then systematically scanned and each green peak has been inspected for surrounding sequences to detect transcription markers. We have used as a basis an automatic TAP+/TAP – ratio score at the target position representing the first base coverage of TAP+ reads divided by the TAP– coverage of the first base (normalized by the number of total reads) (Supplementary Tables S2, S3). We then combined several additional criteria for validation of a potential TSS. These include the presence of well-located promoter motifs upstream of TSS with *in silico* promoter prediction score (see paragraph on *in silico* prediction of promoter motifs associated with different sigma factors), whole transcript RNA-seq data for transcript coverage, differential expression in comparative transcriptome analysis between strain inactivated for a given sigma factor and wild type strain when available.

A total number of 1288 protein-coding genes could be associated with a potential TSS through our analysis. The genomic position of all identified TSSs with corresponding genes is listed in Supplementary Table S2. A large proportion of these TSS corresponds to the first gene of *C. difficile* operons. We have then included the data on possible operon structure for transcriptional unit organization analysis from available gene annotation (Vallenet et al., 2020) and whole RNA-seq expression profiles leading to a total number of more than 2000 genes covered by our TSS mapping (Supplementary Table S2). Additional genes missing in our analysis could be expressed under conditions different from those used in this study, and their TSS identification would require specific expression conditions.

As an example of a large gene cluster, Supplementary Figure S2 depicts the operon map and the promoters of the genes coding for flagella biosynthesis and function. Both one SigA-dependent and three alternative sigma factor SigD-specific promoters could be associated with TSSs for flagella genes (Supplementary Table S2 and Supplementary Figure S2). In the *C. difficile* 630 genome, three loci encode flagellum-associated proteins : late stage flagellar genes *CD0226-CD0240*, flagellar glycosylation genes *CD0241-CD0244* and early stage flagellar genes *CD0245-CD0272* (Stabler et al., 2006; Aubry et al., 2012; Stevenson et al., 2015). The hierarchy of flagellar transcription starts with early stage *CD0245*/*flgB* flagellar operon that contains genes for assembly of the basal body and for the flagellar alternative sigma factor SigD that activates the transcription of the late stage operons. These late stage flagellar genes are involved in assembly of the flagellar hook, filament, and cap, and for post-translational modification of the flagellar filament. We compared our results of TSSs and associated promoter identification with previously suggested transcriptional organization of flagellar cluster and completed its TSS map (Supplementary Figure S2 and Supplementary Table S2, El Meouche et al., 2013; Stevenson et al., 2015). In accordance with previous data, we identified a TSS associated with a SigA-dependent promoter for early stage *flgB* operon and TSSs associated with SigD-dependent promoters for late stage genes *CD0226* encoding putative lytic transglycosylase, *flgM (CD0229)* encoding flagellar anti-sigma factor and *fliC (CD0239)* encoding flagellin (Supplementary Table S2). Additional TSS could be defined for *CD0241, fliQ (CD0261)* and *flgG (CD0269)* genes. Similarly, to the previously reported antisense TSS in the flagellar biosynthesis cluster of *Legionella pneumophila* (Sahr et al., 2012), several TSS could be found in antisense orientation to flagellar genes corresponding to CD630_n00050 – CD630_n00120 antisense RNAs (Soutourina et al., 2013, Supplementary Figure S2 and Supplementary Table S3).

To further validate our approach for TSS mapping, we compared our data with about 35 TSSs previously mapped by 5′ rapid amplification of cDNA ends (5′RACE) or 3′/5′ RACE approach (Table 2). We also performed 5′RACE experiments that unambiguously identified TSSs for two additional genes (*sinR* encoding a transcriptional regulator and *tcdC* encoding protein negatively controlling toxin gene expression). Almost all these TSSs were in agreement with the TSSs identified by 5′-end RNA-seq with only minor deviations in case of multiple possible TSS and/or potential cleavage sites detected. These results and the TSSs already characterized upstream potential ncRNA genes confirmed that we have obtained a robust dataset that describes the transcriptional map of the *C. difficile* strain 630 (Supplementary Table S3, Soutourina et al., 2013).

**TABLE 2:** Comparison of transcriptional start positions identified by RACE and TSS mapping by 5′-end RNA-seq.

Gene	5′ RACE position	5′-end RNA-seq 5′ position	Orientation	Promoter	References
*tcdC* CD630_06640	T 805066	T 805066	-		This work
	A 805037				
	T 805031				
	C 805030				
	A 805028				
	A 805027				
	A 805022				
	T 805505				
	T 805503				
	A 805481				
*tcdR* CD630_06590	A 784644	A 786444	+	P-SigD	El Meouche et al., 2013
	A 786446				
	T 786504				
	A 786505			P-SigA	Dineen et al., 2010
	A 786379				
	A 786239				
*sinR* CD630_22140	G 2566722	G 2566722	+	P-SigA	This work
*sigH* CD630_00570	A 82971	A 82971	+	P-SigA	Saujet et al., 2011
*spo0A* CD630_12140	T 1412457	G 1412458	+	P-SigA	Saujet et al., 2011
	G 1412532	A 1412531	+	P-SigH	
*spoIIAA* CD630_07700	T 942481	G 942480	+	P-SigH	Saujet et al., 2011
CD630_24920	A 2877271	not mapped	−	P-SigH	Saujet et al., 2011
*dnaG* CD630_14540	A 1682862	not mapped	+	P-SigA	Saujet et al., 2011
*sigA2* CD630_14980	G 1734857	A 1734856	+	P-SigH	Saujet et al., 2011
*sigG* CD630_26420	T 3050599	A 3050598	−	P-SigF	Saujet et al., 2011
CD630_02260	G 292850	G 292850	+	P-SigD	El Meouche et al., 2013
*flgM* CD630_02290	A 294568	A 294568	+	P-SigD	El Meouche et al., 2013
*fliC* CD630_02390	G 300874	G 300874	+	P-SigD	El Meouche et al., 2013
CD630_35270	G 4122554	G 4122554	−	P-SigD	El Meouche et al., 2013
CD630_23440	A 2713978	A 2713981	−	P-SigA	Dineen et al., 2010
*glgC* CD630_08820	A 1059909	T 1060072	+		Dineen et al., 2010
*hisZ* CD630_15470	around 1795492	A 1795496	+	P-SigA	Dineen et al., 2010
*cdsB* CD630_32320	A 3784442	A 3784442	−	P-SigL	Gu et al., 2018
*cwpV* CD630_05140	A 607231	A 607231	+	P-SigA	Emerson et al., 2009
*dltD* CD630_28540	A 3337889	A 3337889	−	P-SigA/P-SigV	Woods et al., 2016
	A 3337884			P-SigA/P-SigV	
CD630_25171	T 2908422	T 2908422	−	P-SigA/P-SigB	Maikova et al., 2018
	A 2908421				
CD630_29071	A 3398599	A 3398599	−	P-SigA/P-SigB	Maikova et al., 2018
CD630_09562	A 1124042	A 1124042	+	P-SigA/P-SigB	Maikova et al., 2018
SQ1781 (RCd8)	T 2907991	A 2908006	+		Maikova et al., 2018
	T 2908066	T 2908013			
	A 2907896				
CD630_n00370 (RCd10)	A 1124339	A 1124339	−	P-SigA/P-SigB	Maikova et al., 2018
CD630_n01000 (RCd9)	A 3398302	A 3398302	+	P-SigA/P-SigB	Maikova et al., 2018
CD630_n00860	A 2908058	A 2908058	−		Maikova et al., 2018
SQ2025	A 3306816		−		
	T 3306807	cleavage T 3306807			Soutourina et al., 2013
	A 3306797				
	G 3306788				
CD630_n00210 (RCd4)	A 655066		+		Soutourina et al., 2013
	A 655075	A 655072			
		G 655119			
CD630_n00680 (RCd5)	A 2285913	A 2285913	+		Soutourina et al., 2013
SQ1002	A 1761105	A 1761105	−		Soutourina et al., 2013
	T 1761106				
		T 1760987			
		T 1761212			
SQ173	T 308770	T 308776	+		Soutourina et al., 2013
SQ1498	C 2441928	T 2441927	−		Soutourina et al., 2013
	A 2441933				
CD630_n00030 (RCd2)	T 241079	A 241078	−		Soutourina et al., 2013
	A 241065				
	T 241067				
CD630_n00170 (RCd6)	A 560340	A 560340	−		Soutourina et al., 2013
	C 560320				

The analysis of the nucleotide composition of TSS identified in this study revealed a strong selection for purine with the majority of A (75%) generally required for efficient initiation of transcription by RNAP and to a lower extent of G (14%) and T (9%) (Supplementary Figure S3). The TSS mapping also allowed us to clarify the position of translational start sites for 10 genes. Indeed, the translational start sites of several genes are located upstream of the TSSs suggesting a mis-annotation for translation initiation. We carefully checked the CDS and identified start codons (ATG, TTG or GTG) with a ribosome-binding site located upstream. For example, we modified the translational start sites for the *CD0341, CD1088, CD2055, CD2064, CD2112*, *CD3271* genes encoding conserved proteins of unknown function, *CD1015* and *CD1099* genes encoding two-component system response regulator or histidine kinase, *CD1234* encoding a small protein contributing to *skin* element excision during sporulation and *msrAB* (*CD2166)* gene encoding peptide methionine sulfoxide reductase. The modified annotations are available on MaGe MicroScope platform (Vallenet et al., 2020).

The 5′ untranslated region (5′UTR) also called mRNA leader region located between TSS and translation initiation codon constitutes often a target for important regulatory processes. This region could carry conserved motifs for RNA-based regulations or binding sites for regulatory proteins that could be involved in various transcriptional and post-transcriptional regulatory mechanisms including repression or attenuation of transcription, mRNA stabilization and degradation, riboswitch-based premature termination of transcription and control of initiation of translation. The length of these regions is usually related to their functional importance in gene expression regulatory processes. We analyzed the length distribution of the 5′UTR of the genes with identified TSS (Supplementary Figure S4). For the majority of *C. difficile* mRNAs (75% of the TSSs mapped), the first predicted translational start lies within 100-bp region downstream from the TSS. A total of 392 genes had a TSS located within 20–40 bp from the translational start site, and only few potentially leaderless genes (15) could be found. The presence of leaderless mRNAs has been reported in prokaryotes associated with atypical mechanisms of translation initiation (Jager et al., 2009; Nakagawa et al., 2010). By contrast, 137 mRNAs had leaders longer than 300 bp that could be associated with the presence of particular regulatory motifs. Most of these genes are regulated by specific riboswitches. For example, a number of long-leader containing genes are associated with specific T-boxes responsive to the level of tRNA aminoacylation, among them are genes encoding specific tRNA synthetases, e.g., *serS1, metG, proS, valS* and *asnC*, as well as amino acid biosynthesis and transport genes, e.g., *trpP, leuA, thrC* and *argH*. The large *flgB* flagella operon is associated with a 496-bp 5′UTR region carrying a c-di-GMP-responsive riboswitch (Sudarsan et al., 2008; Soutourina et al., 2013) and a “flagellar genetic switch” that controls the phase variable production of flagella (Anjuwon-Foster and Tamayo, 2017).

### Identification of Two Promoters Upstream Genes and Internal Promoters

For a total of 83 genes, we detected the possible existence of two TSSs (Supplementary Table S2 column A highlighted in green, Figure 1A, Supplementary Figure S5A). In the majority of cases, these additional TSSs were associated with lower signal from deep-sequencing data and either the same or a different sigma factor recognition motif could be found upstream of the TSSs. We could hypothesize that for these genes two alternative promoters could be used depending on the environmental factors and growth conditions. For example, *rpmH (CD3680)* gene encoding 50S ribosomal protein L34 is associated with one SigF- and one SigA-dependent promoter having different deep-sequencing signal intensity for both 5-end RNA-seq and whole transcript RNA-seq (Figure 1A). Likewise, for *cbpA (CD3145)* gene encoding surface-exposed adhesin, a first TSS potentially associated with SigA-dependent promoter is detected with lower signal intensity while a second SigA-dependent TSS is revealed with higher sequence reads number (Figure 1A). Interestingly, at least two potential cleavage sites could be found in 5′-end of *cbpA* CDS with high intensity signal from both TAP-treated and non-treated samples. In addition, potential internal TSS could be detected inside the coding part of this gene (Figure 1A). Two different promoters associated with different sigma factors were detected upstream of the *CD0761* gene encoding a putative ATP-dependent RNA helicase and *mreB (CD1145)* gene encoding rod-shape determining protein MreB (Supplementary Figure S5A). In these cases, either a SigF- or a SigH-dependent promoter is present in addition to a SigA-dependent promoter (Supplementary Figure S5A and Supplementary Table S2).

A number of internal TSSs in the coding regions of genes was also detected, which further demonstrated a complex transcriptional architecture of *C. difficile* genome (Supplementary Table S2, column I highlighted in blue, Figure 1B). In many cases, these internal TSS map to the genes with identified primary TSS in accordance with the mechanisms for internal transcription initiation by elongating RNAP complexes suggested in bacteria (Shao et al., 2014; Cuklina et al., 2016; Harden et al., 2016). Moreover, internal transcription initiation could be at the origin of 3′-end derived ncRNA as recently demonstrated in other bacterial species (Chao et al., 2012; Guo et al., 2014). In addition to a primary TSS identified upstream of the *CD0156* gene, we could find a TSS associated with −10 box for SigA-dependent promoter in the 3′-end of this gene encoding a putative membrane protein while no known downstream gene could be identified in sense orientation (Figure 1B). Similarly, the TSS for *coaE (CD1129)* gene encoding dephospho-CoA kinase lies in the middle of the upstream *polA (CD1128*) gene encoding DNA polymerase I (Supplementary Figure S5B). In addition to primary TSS upstream of *CD3045* gene encoding putative ATPase, two internal promoters could be identified inside this gene that could drive the transcription of downstream *CD3044* gene encoding a transcriptional regulator of the RpiR family (Supplementary Figure S5B). This is an example of internal TSS that could be found inside the upstream gene co-transcribed with the downstream gene within the same operon. The presence of these internal promoters might allow to change the ratio of protein production of co-transcribed genes, a differential expression of genes inside operons or more complex regulatory processes related to alternative operon structure and co-transcriptional relationship between genes (Sorek and Cossart, 2010; Shao et al., 2014).

### Identification of Cleavage Sites Within Transcripts

In addition to TSS mapping, the comparison of RNA-seq signal intensity between TAP+ and TAP− samples allowed us to suggest several potential cleavage sites corresponding to positions with large number of reads in both samples treated or not with TAP enzyme (TAP+/TAP− ratio close to 1), located downstream from identified TSS and not associated with promoter consensus sequences from bioinformatics search. Examples of these sites potentially cleaved by cellular ribonucleases are shown in Figure 2 and Supplementary Figure S6. Different ribonucleases such as RNase III, RNase Y or RNase J could be involved in these potential processing events and could recognize particular secondary structures rather than specific sequence motifs (Trinquier et al., 2020). In the majority of cases, the potential cleavage sites are situated just downstream from the TSS at 23–62 bp and up to 87 bp distance and from 0 to 54 bp upstream of translation initiation RBS site (Figure 2). The processed mRNA would thus retain in the majority of cases the RBS and codon for translation initiation. For the *CD0564* gene encoding ATP-dependent protease, the *modA (CD0869)* gene encoding molybdenum ABC-type transporter, the *gcvTPA (CD1657)* glycine decarboxylase operon and the *CD2396* gene encoding conserved protein of unknown function the potential cleavage site is situated at 0 to 3 bp from RBS (Figure 2). By contrast, for the *CD1392* gene encoding a putative ribonuclease, the potential cleavage site lies at “A” position inside the “GGAGG” RBS (Supplementary Figure S6). Sometimes, it is difficult to distinguish between potential cleavage sites and the presence of additional TSS based on TSS mapping data visualization (Figure 2, Supplementary Figure S6 and data not shown). Conversely, a part of potential TSS (Supplementary Tables S2, S3) could correspond to cleavage sites. A complex profile (Supplementary Figure S6) could be observed with several TSS and potential cleavage sites associated with the same gene, e.g., for the *CD3145* adhesin-encoding gene (Figure 1A). *CD1329* gene encoding RNaseY, *CD1392* gene encoding a putative ribonuclease and *ptsH* (*CD2756*) gene encoding HPr protein of PTS system also presented a complex profile with several potential cleavage sites inside the gene (Supplementary Figure S6). Interestingly, a potential cleavage site could be also detected in the 3′-part of the *agrB* gene encoding accessory gene regulator of a quorum-sensing system (Supplementary Figure S6). This gene is co-transcribed with autoinducer prepeptide *agrD* gene. The cleavage of the bicistronic *agrB-agrD* mRNA will result in an *agrD* transcript and could lead to the stabilization of the processed transcript.

### Transcription in the Pathogenicity Locus and Key Regulatory Genes

In *C. difficile*, the main virulence factors, toxins TcdA and TcdB, as well as toxin expression regulatory components TcdR, TcdC and TcdE are encoded in the 19.6 kb chromosomal region, called pathogenicity locus (PaLoc) (Braun et al., 1996; Martin-Verstraete et al., 2016). TcdR is an alternative sigma factor (Table 1) driving the transcription of *tcdA*, *tcdB* and *tcdR* genes, while TcdC negatively controls toxin gene expression by interfering with transcription initiation by TcdR-containing RNAP (Mani and Dupuy, 2001; Matamouros et al., 2007). Despite a relatively low level of PaLoc region gene expression, the analysis of sequencing data allowed us to define a TSS for both *tcdR* and *tcdC* regulatory genes, that were in accordance with RACE analysis previously published for *tcdR* (El Meouche et al., 2013) or performed in this study for *tcdC* (Table 2). The absence of detection of TSSs upstream of *tcdA* and *tcdB* that have been previously mapped (Dupuy and Sonenshein, 1998) might be due to the low level of transcription of these genes under the conditions used.

Figure 3 and Supplementary Figure S7 show representative examples of TSS mapping for several genes encoding regulators such as CcpA, CodY, SigH, RstA, LuxS, LexA, and SinR known to directly or indirectly control toxin gene expression (Martin-Verstraete et al., 2016). Most of these regulatory genes are driven by SigA-dependent promoters. In addition to primary TSS upstream of the *codY* gene, a second TSS, which is potentially associated with a SigF-dependent promoter could be identified inside the upstream gene (Supplementary Figure S7). The comparison of TAP-treated and non-treated samples also suggested the presence of potential cleavages sites just downstream from the TSS of *ccpA* and *lexA* genes, and in the 3′-end of *codY* and *fur* genes. Interestingly, a cleavage site could be detected between *cggR* (*CD3175*) and *gapA* (*CD3174*) genes as observed for the polycistronic *cggR-gapA* operon maturated by RNase Y in *B. subtilis* and *S. aureus* (DeLoughery et al., 2018) (Figure 3). This processing event could also lead to a differential stability and thus discordant abundance of co-transcribed genes encoding glycolytic enzyme and its transcriptional regulator in *C. difficile*.

### *In silico* Prediction of Promoter Motifs Associated With Different Sigma Factors

14 genes encoding sigma factors (two copies of SigA, SigH, SigF, SigE, SigG, SigK, SigB, SigD, TcdR, SigV, CsfU, CsfT and SigL) are present in the *C. difficile* 630 genome (Table 1). In addition, eight potential sigma factors associated with mobile genetic elements mainly in conjugative transposons of Tn916, Tn1549 or Tn5397 family are annotated in the genome (Sebaihia et al., 2006; Brouwer et al., 2011). These proteins might be involved in the transcription of genes inside these mobile genetic elements but we cannot exclude that they also contribute to expression of genes outside the transposons. The relatively high number of sigma factors suggests a very complex pattern of transcription in *C. difficile*. To characterize potential promoter consensus sequences, we analyzed the nucleotide sequences upstream of the TSSs. We extracted the 100 nucleotides (from −99 to +1) upstream of the TSS for protein-coding genes and ncRNA genes and analyzed them for the presence of motifs previously identified for a smaller set of *C. difficile* promoters under the control of SigB, SigH, SigE, SigF, SigG, SigK, TcdR and SigD or *B. subtilis* promoters (SigA, SigL) (Mani et al., 2002; Saujet et al., 2011, 2013; El Meouche et al., 2013; Kint et al., 2017). By contrast, consensus sequences for the three ECF sigma factors and for the putative sigma factors associated with mobile genetic elements in *C. difficile* remain to be identified and we cannot search for the corresponding promoters upstream of TSS.

**FIGURE 4:**
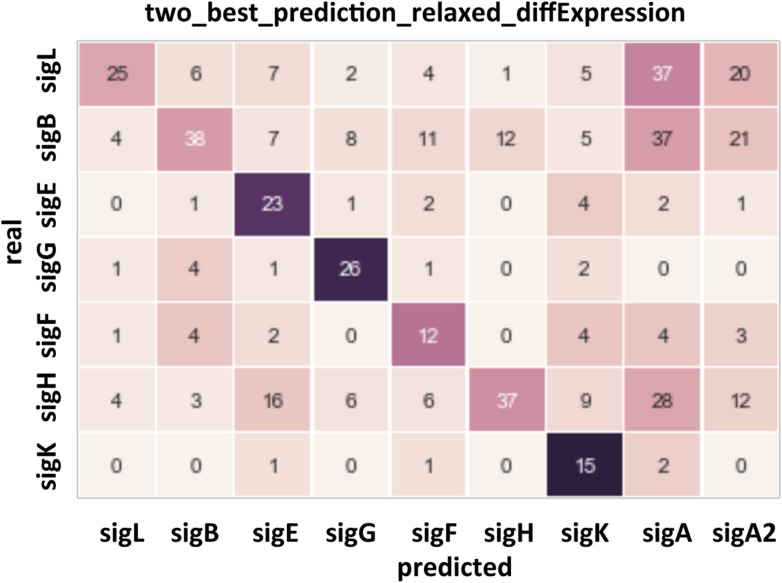
Confusion matrix for the prediction of the sites recognized by the main sigma factors of *C. difficile*. Rows: actual sigma factors, based on changes in gene expression in knockout mutants. Columns: predicted sigma factors. A prediction was listed as correct if the actual sigma factor was among two best ones, and incorrect otherwise, in the latter case the top-scoring sigma factor was listed as the prediction.

The automatic search for promoters upstream of TSS is known to be difficult due to variations in the distance between −10 and −35 boxes or between the TSS and the −10 element and sometimes degenerated consensus sequences. The AT-rich nature of *C. difficile* genome especially in intergenic regions (Supplementary Figure S3) also complicates the search for promoters. To identify sigma factors corresponding to TSSs as reliably as possible, positional weight matrices (PWMs) for sigma factors were made on the basis of a set of binding sites in *C. difficile* genome that are identified by comparing expression profiles of WT strain versus mutants inactivated for each sigma factor. Data were previously obtained for SigK, SigH, SigF, SigG, SigD, SigE, SigB and TcdR (Mani et al., 2002; Saujet et al., 2011, 2013; El Meouche et al., 2013; Fimlaid et al., 2013; Kint et al., 2017). Concerning SigL, we performed a transcriptomic analysis (see below). For SigA, the consensus was based on *B. subtilis* sites from database of transcriptional regulation in *B. subtilis* (DBTBS) (Sierro et al., 2008). After training (Supplementary Figure S8), we applied the obtained positional weight matrices to genes that have demonstrated differential expression in mutants for each sigma factor. The results are shown in Figure 4 and Supplementary Table S4. The predictions for sporulation specific sigma factors (SigF, SigE, SigG, SigK) largely matched the experimental data but allowed to slightly extend each regulon (see below). The automatic predictions for sigma factors with larger regulons (SigL, SigB, SigH) were relatively less reliable. In particular, SigA was predicted as the main factor for many of them. It could be caused by two reasons. Firstly, these promoters could be recognized by two sigma factors, SigA and another sigma factor. Secondly, some genes could indeed be transcribed only by SigA, and their expression could change in the other *sig* mutants due to downstream global effects on other genes involving regulatory cascades.

The motifs for SigA-dependent promoter could be found closely upstream of TSS for 743 genes in *C. difficile* genome as best prediction (Supplementary Tables S2, S3). An extended −10 box with consensus sequence TGNTATAAT could be identified upstream of 245 TSSs (Supplementary Tables S2, S3) (Browning and Busby, 2016). For these genes, no −35 consensus or only a weak consensus could be usually found in promoter region. Extended -10 promoters do not require a strong −35 sequence for efficient transcription (Keilty and Rosenberg, 1987; Gruber and Gross, 2003). Indeed, the extra TG dinucleotide stabilizes open complex formation by providing critical contacts with region 3.0 of RNAP (Gruber and Gross, 2003; Murakami and Darst, 2003). In addition, for several promoters, it is difficult to discriminate between extended −10 promoters and SigB-dependent promoters using the consensus recently defined in *C. difficile* (Kint et al., 2017) (Supplementary Tables S2, S3, S4). Finally, some genes controlled by transcriptional activators have only weak consensus motifs at −35 position or even no −35 box and we found a series of promoters with only a −10 box (Supplementary Tables S2, S3, S4).

**FIGURE 5:**
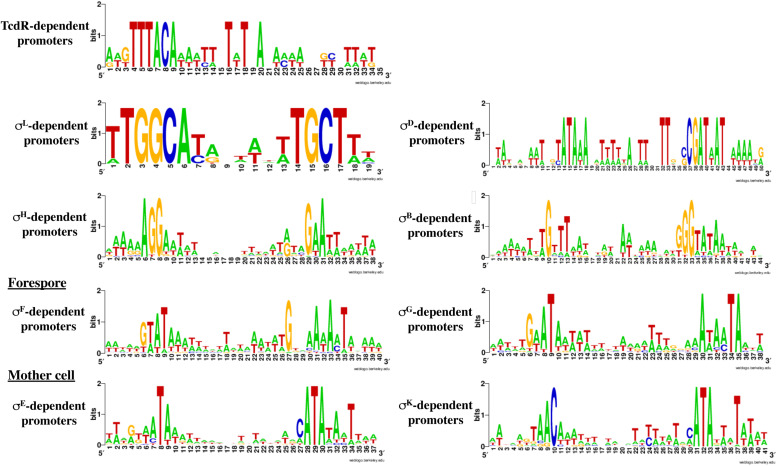
Consensus sequences for promoters recognized by the different sigma factors. These consensus were generated by WebLogo using the data presented in Supplementary Tables S2, S4–S6 and Table 3. The consensus for TcdR (Table 1) was obtained from characterized promoters (Mani et al., 2002; Martin-Verstraete et al., 2016).

Based on the PWMs, previously reported transcriptomic data and manual inspection of promoters, we assigned a corresponding sigma factor involved in the transcription for most of the genes (Supplementary Tables S2, S3) and we refined the respective consensus sequences of promoters recognized by individual RNAP associated with specific sigma factors (Figure 5). We will discuss below in more detail the results obtained for SigH, the sigma factors specific of sporulation and SigL.

In accordance to our previous analysis (Soutourina et al., 2013), the majority of ncRNA genes could be associated with SigA-dependent promoters. Upstream of a few ncRNA TSS, we predicted the presence of promoters probably recognized by SigD, SigB or sporulation-specific sigma factors (Supplementary Tables S3, S4).

### Genes Transcribed by Sigma Factors of the Transition Phase and of Sporulation

**FIGURE 6:**
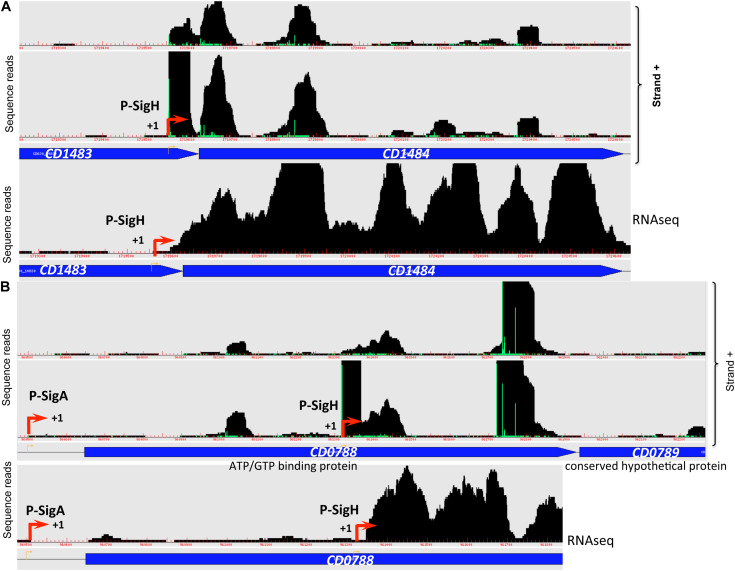
Promoters controlled by SigH in *C. difficile.* Examples of 5′-end RNA-seq (TAP–/TAP+ profile comparison) and RNA-seq data for dual tandem TSSs and/or internal TSSs inside coding sequences corresponding to SigH-dependent promoters are shown. Panel **(A)**: *CD1482-CD1484* operon and panel **(B)**: *CD0788-CD0789* operon.

Transition from exponential growth toward stationary phase represents a key checkpoint in *C. difficile* development associated with the induction of toxin production and decisions to initiate sporulation or biofilm formation. By comparing the transcriptome of the *C. difficile* strain 630Δ*erm* and its isogenic *sigH* mutant, we have previously identified 286 genes, which are positively controlled by SigH (Saujet et al., 2011). To identify genes likely transcribed by RNAP associated with SigH, we combined the transcriptome analysis previously published (Saujet et al., 2011) and the *in silico* analysis of promoters (Table 3, Supplementary Tables S2, S4). We can then propose a list of 41 genes down-regulated in transcriptome in the *sigH* mutant with a consensus for SigH-dependent promoters identified as best hit (Table 3). Two promoters having a lower score of promoter identification were not used to refine the consensus (Figure 5). 12 additional genes with a consensus for SigH as best hit are not controlled by SigH in transcriptome in our conditions, two of these genes having a second mapped promoter. In addition to genes involved in sporulation (*spo0A*, *spoIIAA*, *spoVD*, *spoVS, spoVG* and *CD0572*) (Saujet et al., 2011) and cell division (*minC*, *ftsZ*, *CD2650* and *CD3673*) (Saujet et al., 2011), we found genes involved in translation (*fusA1*, *frr*) (Sebaihia et al., 2006), in envelope-related processes (*cwp27*, *CD0573*, *lplA, CD3458*) (Sebaihia et al., 2006), in metabolism (*CD0865*, *fdhF, CD0999*, *CD1484* and *CD2989*) (Neumann-Schaal et al., 2019) and genes encoding 11 conserved hypothetical proteins of unknown function. Interestingly, two genes of the *CD0999* and *CD2989* operons encoding sulfonate ABC transporters as well as a gene *CD1484* encoding a sulfonate binding protein were expressed under the control of a P-SigH promoter. This suggests that these sulfur-containing compounds play a key role at the onset of stationary phase as alternative sulfur-sources. It is worth noting that *ssuA* is the last gene of an operon encoding another sulfonate ABC transporter. The first two genes of this operon were not controlled by SigH. We detected an internal promoter located within *CD1483* with a consensus recognized by SigH in agreement with the specific transcriptional control of *ssuA* by SigH and the increased transcript level observed for this gene in RNA-seq (Figure 6A). An internal promoter recognized by SigH is also present upstream of *CD0789* in the *CD0788-CD0789* operon (Figure 6B).

**TABLE 3:** Promoters transcribed by SigH associated to the RNAP.

Gene	Function	Expression ratio^&^ *sigH*/630Δ*erm*	Promoter recognized by SigH	Score promoter
*CD0022 fusA1*	Elongation factor G	0.04	AAA**AGAGGACAA**TTACCTCCAGAT**GTAGAAAT**AAAGCTAGTA	4.29
*CD0142#*	RNA-binding protein	0.14	AAA**AGAGGATTA**TGAGAGTTCGT**GTAGAATA**TATTATTA	4.30
*CD0148*	Conserved hypothetical protein	0.42	GAA**AAAGGTATC**TACAATATACAG**AGCGAATT**TATAATA	4.16
*CD0440 cwp27*	Cell wall binding protein	0.25	CAT**AAAGGAAAA**TATTCTTTTAT**GTAGAATTA**TAATATTTAAGTAA	5.82
*CD0572#*	Sporulation protein	0.49	GAT**AAAGGTATT**TGGAAAAGTAT**GAAGAATAC**AGAAATAGA	3.64
CD0573*	Membrane protein	0.45	ATA**AAAGCTATC**CATATAAAATT**AAAAAAATT**CTTTTTAA	1.57
*CD0770 spoIIAA*	Anti-SigF factor	0.03	ATT**GAAGGAATA**AAAATATAATT**ATAGAATT**GATTAAAAG	4.93
*CD0789*	Conserved hypothetical protein	0.3	AAT**GCAGGAAAA**TCTACCCTGTTG**AACGAATT**AATAAAGA	4.02
*CD0838*	DNA-binding protein	0.26	GTA**CAAGGATTT**TAAAGATAAAT**ATAGAAAT**TAATGTTTA	4
*CD0865*	ADP-ribose binding protein	0.08	TTA**ACAGGATTT**ATGTAGGTGTTT**ATAGAAAT**AAATAAATA	4.7
*CD0999*	ABC-type transport system, sulfonate/taurine	0.35	AAA**GAAGGATTA**TTCGATAAATTA**ACAGAAAT**TAGTATAAA	4.3
*CD1149 minC*	Cell division regulator	0.09	ATA**AAAGGGTTT**AAAGCGTATTT**GAAGAATA**TAAATGA	3.48
*CD1214 spo0A#*	Stage 0 sporulation protein A	0.2	TTA**GGAGGAATA**TAATTTTGGAGT**GTCGAATA**TGCTTTA	5.79
*CD1221*	Membrane protein	0.5	ATA**AAATGAATA**ACCCTTTGTCCT**AAAGCATA**CTATTTTATA	3.41
*CD1264*	Conserved hypothetical protein	0.08	AAA**AGAGGAAAA**ATCATTTTAAT**GTAGAATA**ATTGTACA	5.2
*CD1317*	Conserved hypothetical protein	0.42	TTA**AAAGGAAAG**ACAGGATAAAT**ATAGAAAT**TTTATAACA	4.55
*CD1484 ssuA*	ABC-type transport system, alkanesulfonates family	0.08	ATA**AAAGGATTA**AACAATTTAGT**GTCGAAGA**TGAATATAA	4.42
*CD1498 sigA2*	RNA polymerase sigma factor	0.05	ATA**TAAGGAGGA**TATTGCTGTTA**GAAGTAGA**ATAATATTATA	4.74
*CD1543.1*	Conserved hypothetical protein	0.07	TAT**AAAGGAAAA**ACCTCTTTTAAT**GTAGAAAC**TTATATTG	5.4
*CD1622#*	Conserved hypothetical protein	0.46	AAA**AGAGGCTTG**TTTCCATTTTGAG**ACAGTATA**ATTTTTATGTA	2.09
*CD1654 lplA*	Lipoate-protein ligase	0.34	TTT**AAAGGAGTT**TTTATATTATT**GTCGAATT**ATAAAATTA	5.43
*CD1878*	Sigma factor Tn1549-like	0.45	GAT**GGAGGAAAC**TTTATGGCAAA**AGAGTATT**ATCTTTATATCA	2.73
*CD1900*	Conserved hypothetical protein	0.12	ATA**AAAGGAATT**TACAAGTTATGT**GTTGTATA**GATATTGTA	2.54
*CD1935 spoVS*	Stage V sporulation protein S	0.03	AAT**AAAGGTTTT**CTTAAAACGATT**ATAGAAGT**ATATTTTTA	4.24
*CD1941*	Conserved hypothetical protein	0.05	TAA**CAAGGAAAC**AGAACCCTCTC**ATAGAAAT**TAAATTATTA	3.68
*CD1967*	Conserved hypothetical protein	0.03	TTA**GAAGGAAAA**TAGCTTTTATC**ATCAAATT**AATAATTA	5.08
*CD2063*	Conserved hypothetical protein	0.45	GAT**AGAGGATTT**ATAAGTGTTTAA**GGTGAATT**AAATATAA	3.48
*CD2137 frr*	Ribosome-recycling factor	0.41	AAT**AAAGGTATT**TGAGCTTACAAC**AGAGAATA**TAATAAGA	3.62
*CD2447*	Putative histidine triad protein	0.06	GTA**GAAGGAATT**TTGCTATAACAT**GTAGAAAT**TATAATATTTA	4.83
*CD2646 ftsZ*	Cell division protein FtsZ	0.34	TAA**AAAGGAAAA**TTTACGTTTTT**GTGGAATA**TGTTACTTA	4.57
*CD2650*	Cell division protein Fts-Q type	0.34	TTT**GTAGGAAAA**ACAAGCTCTAAA**GGTGTATT**TATTAACCA	2.66
*CD2656 spoVD*	Stage V sporulation protein D	0.15	TCT**AAATGAATA**TAAAAATAAAAA**AAAGAATA**ATTATAAAAA	3.58
CD2657*	Conserved hypothetical protein	0.36	TTT**AGAGGAAGA**AATATGATTAAT**AACAAATA**TAGTATA	1.82
*CD2989 ssuA2*	Sulfonate-family ABC-type transporter	0.39	AAA**GAAGGATTA**AGTATGGATGAT**GTGGAATT**TGTTAATA	4.6
*CD3221*	Peptidase, M20D family	0.16	GAA**ACAGGGGAA**TTATTATTAAT**GGTGAATT**ATTTAATA	2.85
*CD3290*	Conserved hypothetical protein	0.19	ACA**AGAGGGATT**GTTGATGATTTT**ATCGAAAT**CCTAATTA	5.02
*CD3317 fdhF*	Formate dehydrogenase-H	0.31	TTA**AGAGGAATT**GTGAGAAAATT**GTTGAATT**TAATAGATA	3.76
*CD3458*	Membrane protein	0.07	GTT**AAAGGAAAT**TGTAGGTAGTTT**ATCGAATT**GTTTAATCA	5.13
*CD3516 spoVG*	Regulator of spore cortex synthesis	0.01	AAA**AGAGGATAT**CCCTAGTTGTTC**ATAGAATT**ATTTA	4.89
*CD3673*	DNA-binding protein Spo0J-like	0.21	TTC**TCAGGAAAT**AATTAAGCTTACT**GTAAAAAA**ACAA	2.16

# means that two promoters are mapped upstream of the gene. *promoters with a lower score not used to obtain the consensus, which was determined with the promoters with a score > 2. & as determined by transcriptome analysis (Saujet et al., 2011). The genes underlined have been previously mapped by RACE (Saujet et al., 2011). The TTSs mapped are underlined and the −10 and −35 boxes are indicated in bold.

Spore formation is a tightly controlled process that constitutes an essential step in *C. difficile* life cycle for its dissemination and survival. We have previously compared our TSS mapping data (Soutourina et al., 2013) with transcriptomic data done with microarrays comparing wild-type strain with mutants inactivated for *sigF*, *sigE*, *sigG* or *sigK* genes encoding major sporulation sigma factors at different times during sporulation in liquid media (Saujet et al., 2013). We now extended this comparison to RNA-seq data obtained using RNA extracted after 18 h of growth on plates by Aimee Shen and co-workers (Fimlaid et al., 2013; Pishdadian et al., 2015). Even if the microarray and RNA-seq data largely overlapped, this new analysis allowed us to propose a more complete list of genes transcribed by the sigma factors of the forespore, SigF and SigG (Supplementary Table S5) or the mother cell, SigE and SigK (Supplementary Table S6) and to refine the consensus of the promoters recognized by each of these sigma factors (Figure 5). We identified 10 new SigE-dependent promoters (*CD0557*, *CD0629*, *CD1022*, *CD1043*, *CD1403*, *CD1575*, *CD1629*, *CD2316*, *CD2443*, *CD3466*) while only two additional SigK-dependent promoters (*CD2167* and *CD2720*) can be proposed (Supplementary Table S6). For *glmS* and *CD1340* that are controlled by SigK, both a SigK- (best hit) and a SigA-dependent promoter are detected upstream of these genes. In addition, for four genes (*CD1511*, *CD1930*, *CD2443* and *CD3350*), the best hit detected *in silico* did not fit with the transcriptome data. In the forespore (Supplementary Table S5), we identified 56 promoters recognized by SigF and /or SigG, including 12 new ones compared to our previous analysis (Saujet et al., 2013). 19 and 16 genes can be assigned to the SigF or the SigG regulon, respectively. 15 additional promoters have the key motifs recognized by SigF (Supplementary Table S2) but we failed to detect forespore-dependent control (SigF- or SigG-dependent) of their expression. For six of them, this absence of control might be due to the presence of a second promoter upstream of the gene. For 21 additional forespore-controlled genes, our data are ambiguous (Supplementary Table S5). Although a consensus closer to those recognized by SigG than by SigF was identified upstream of the TSS, the expression of *CD2266*, *CD2599* and *CD2856* was only controlled by SigF. Unexpectedly, *CD1789*, *CD2245.1* and *sigG* were controlled by SigG but not by SigF despite the fact that a consensus recognized by SigF is proposed upstream of these genes (Supplementary Tables S5, S4). In *C. difficile*, *sigG* is transcribed from at least two promoters, one in front of *spoIIGA* recognized by SigA, and the other just upstream of *sigG* recognized by forespore specific sigma factors (Saujet et al., 2013). Interestingly, *CD2245.1* is located downstream from the *cspBAC* operon, a member of the SigE regulon and is also less expressed in the *sigE* mutant than in the WT strain in transcriptome contrary to most forespore-controlled genes (Saujet et al., 2013). It is tempting to speculate that a more complex profile of expression with a possible readthrough from *cspBAC* also exists for *CD2245.1*. Finally, 15 genes controlled both by SigF and SigG as expected for members of the SigG regulon have a consensus closer to those of SigF-dependent promoters than SigG-dependent promoters. This included genes encoding 3 SASPs (*CD1290*, *CD3220.1*, *CD3249*), a catalase (*CD1567*), a dipicolinate transporter (*spoVAC* operon) and the SpoVT regulator. The results obtained for *spoVT* strongly suggest that this gene is transcribed by both SigF and SigG (Fimlaid et al., 2013; Saujet et al., 2013). The existence of a residual transcription of *spoIIR* in the *sigF* mutant in the forespore also suggests that SigG might allow *spoIIR* transcription. Due to the promiscuity of the consensus of SigF and SigG-controlled promoters (Figure 5), we can propose a probable existence of an overlapping SigF and SigG regulons.

### SigL-Dependent Promoters in *C. difficile*

SigL is a sigma 54 type sigma factor. In the genome of *C. difficile*, an important number of EBPs is present (Nie et al., 2019). Since EBPs activate SigL-dependent promoters, we can expect a large set of SigL-controlled genes in this enteropathogen. However, SigL and its regulon remain poorly characterized in *C. difficile* despite its possible important role in the control of metabolism. Among the TSS mapped by 5-end RNA-seq and identified as potential SigL targets *in silico* (Supplementary Table S2 and Figure 4), we first searched for the presence of the highly conserved sequence GGC at position −24 and GC at position −12 (Francke et al., 2011). We identified 13 promoters with these conserved motifs upstream of TSSs (Supplementary Table S4 and Table 4). The alignment of these “−24,−12” promoters led to the consensus TGGCA-N_6_-[A,T]TGCT[A,T] (Figure 5). We used this consensus to check for additional “−24,−12” promoters in the 200 pb upstream of start codons of all *C. difficile* genes. By this approach, we identified 30 putative SigL-dependent promoters corresponding to about 95 controlled genes (Table 4). As described in other bacteria (Danson et al., 2019; Nie et al., 2019), it is worth noting that 23 genes or operons with a “−24,−12” promoter out of 30 are adjacent on the chromosome to genes encoding a sigma 54-dependent activator (EBP), which is probably involved in their control.

**TABLE 4:** SigL-dependent promoters in *C. difficile* 630 based on TSS mapping, transcriptome data and *in silico* analysis.

Gene	Operons	Functions	Fold change *sigL::erm*/ 630Δ*erm*	Promoter -24,-12	SigL-dependent associated regulator
*CD0040*	*CD0040-CD0043*	Activator, PTS Galactitol family		A**TGGCA**TATAAG**TTGCTA**T	CD0040, LevR-type
*CD0166*	*CD0166-CD0165*	Peptidase, amino acid transporter	0.17*	A**TGGCA**TAATAA**TTGCTT**A	CD0167, GamR-type
*CD0284*	*CD0284-CD0289*	PTS Mannose/fructose/sorbose family	0.2 to 0.3	T**TGGCA**CGGCAA**TTGCTT**A	CD0283, LevR-type
*CD0395/hadA#*	*hadAIBC-acdB-etfBA1*	Leucine utilization	< 0.001 (0.00001*)	T**TGGCA**CGATTT**ATGCTT**T	CD0402/LeuR
*CD0442/ord*#	*ord-ortAB-oraSEF-orr-nhaC*	Ornithine degradation	NR	T**TGGCA**CGATTT**ATGCTT**T	CD0441/OrdR
*CD0490*#	*CD0490-CD0494*	Sugar-P-dehydrogenase, PTS mannose/fructose/sorbose family	0.24*	T**TGGCAT**GAAAG**TTGCTT**T	CD0516? LevR-type
*CD0800/crt1*#	*crt1*-*CD0801-catB-bcd-etfBA2*	Crotonase, permease, CoA transferase, acyl-CoA dehydrogenase, EtfBA	NR	T**TGGCA**TAGTAC**TTGCTA**T	CD0806/YctR
*CD0860*	*CD0860-CD0863 -malH1*	PTS lactose/cellobiose family- Maltose-6*P glucosidase		C**TGGCA**TAATAC**TTGCTT**A	CD0858°
*CD1187*#	*CD1187-CD1189*	CHP, γ-glutamyl-γ-aminobutyrate hydrolase, amino acid permease	NR	T**TGGCA**TACATA**TTGCTA**A	CD1186 GamR-type
*CD1413/rhaT*#	*CD1413*	Membrane protein (RhaT)	0.31 (0.19*)	A**TGGCA**TAGTTT**TTGCTT**A	CD1412/XhaQ
*CD1555*#	*CD1555*	Putative serine/threonine exchanger	0.36 (0.5*)	T**TGGCA**TAATAT**ATGCTT**A	?
*CD1740*#	*CD1740-CD1741*	Sarcosine reductase complex	NR	T**TGGCA**TAGAAA**ATGCTT**T	CD1739/SarR, TCS SarRS
*CD2091* #	*CD2091-CD2089*	Putative Xanthine/uracile permease (PbuX), adenosine derivate deaminase	NR	T**TGGCA**TTATAA**TTGCTT**C	CD2092-DioR1
*CD2279*	*CD2279*	Sugar-P dehydrogenase		A**TGGCA**TAGATA**TTGCTA**T	CD2283?
*CD2283*	*CD2283-CD2280*	Activator, PTS fructose/mannitol family	NR	A**TGGCA**TGATAG**TTGCTT**A	CD2283 LevR-type
*CD2327#*	*CD2327-CD2323*	Arabitol/xylitol PTS, sugar-P dehydrogenases	0.03*	T**TGGCA**CACAAC**ATGCTT**T	CD2328 LevR-type
*CD2382*#	*CD2382-iorAB-butK*	Aromatic aminotransferase, Indole-pyruvate oxidoreductase, butyrate kinase	0.2 to 0.02 (0.01*)	T**TGGCA**TAGTAA**TTGCTT**A	CD2383/ZypR
*CD2699*#	*CD2699-CD2697*	Membrane proteins, peptidase	0.03 to 0.09 (0.01*)	T**TGGCA**TAAGTT**TTGCTT**A	CD2700
*CD2733*	CD2733-CD2734.1	PLP-dependent transferase, Na^+^/H^+^ antiporter, membrane protein		T**TGGCA**CGTTGT**TTGCTT**A	CD2732
*CD2862*#	*CD2862-CD2860*	dipeptidase, membrane proteins		T**TGGCA**CATCAA**TTGCTA**C	CD2863
*CD2870*/ *kdgT1*	*kdgT1*-*uxaA*°	2-keto 3-deoxygluconate permease, altronate dehydratase	0.12 to 0.23	T**TGGCA**TAGTAA**TTGCTT**T	CD2869/XduR
*CD3093#*	*CD3093*	γ-glutamyl-γ-aminobutyrate hydrolase		T**TGGTA**TGCTAC**TTGCTC**T	CD3094 GamR-type
*CD3094*	*CD3094*	Sigma-54 dependent regulator		T**TGGCA**CAATTT**TTGCTT**T	CD3094 GamR-type
*CD3184/dpaL2*	*dpaL2*	Diaminopropionate amonia lyase	0.3*	T**TGGCA**CGGTAA**TTGCTT**T	CD3186/DioR2
*CD3187*	*tdcF*	Putative regulatory endoribonuclease		T**TGGCA**CGTTAA**TTGCTT**	CD3186/DioR2
*CD2085*	*dpaL1*	Diaminopropionate amonia lyase		T**TGGCA**TGTTAA**TTGCTT**A	CD3186/DioR2
*CD3232*/*cdsB* #	*cdsB*	Cysteine desulfidase	NR^%^	A**TGGCA**TGTATT**TTGCTA**T	CD3233/CdsR^
*CD3244/prdA*	*prdA-CD3243-prdBDE*-*prdE2F CD3236*	Proline utilization	0.4 to 0.5	T**TGGCA**TAGGAA**TTGCTT**A	CD3245/PrdR
*CD3247*/ *prdC*	*prdC*	Proline utilization		T**TGGCA**TAGAAA**TTGCTT**T	CD3245/PrdR
*CD3279*	*CD3279-CD3275*	PTS Mannose/fructose/sorbose family, sugar-P isomerase		T**TGGCA**TACTTT**TTGCTT**T	CD3280 LevR-type

# the transcriptional start sites were mapped by 5′-end RNA-seq (see Supplementary Table S2). * as determined by qRT-PCR. °pseudogen in strain 630. % *cdsB* is controlled by SigL in TY in the presence of cysteine (Gu et al., 2018). ^The CdsR regulator of *C. difficile* is of type 2 (Nie et al., 2019). NR = non regulated by SigL in qRT-PCR in the conditions tested. For most of them, the transcriptional regulators and genes were renamed as proposed by Nie et al. (2019) or by Dannheim et al. (2017).

### SigL-Mediated Control of Gene Expression in *C. difficile*

To identify genes expressed under the control of SigL, we performed a transcriptome analysis comparing the expression in the strain 630Δ*erm* and the *sigL::erm* mutant after 4 h of growth in TY. Approximately 7.5 % of the *C. difficile* genes were found to be differentially expressed between these two strains. 165 genes were up-regulated and 124 genes were down-regulated in the *sigL::erm* mutant (Supplementary Table S7). We confirmed these results by qRT-PCR analyses for 10 genes (Table 4). Only 27 genes identified as containing a “−24,−12” promoter and very likely controlled by SigL were down-regulated according to the transcriptomic data obtained. We were able to detect 4 additional operons (13 genes) as controlled by SigL using qRT-PCR (Table 4). These results are not surprising since most of the genes transcribed by RNAP associated to SigL respond to specific inducers through their associated EBP activators sensing these signals (Francke et al., 2011; Nie et al., 2019). For example, cysteine or proline strongly induces the expression of *cdsB* and *prdC* genes and the *prdA* operon, respectively, in a CdsR- or a PrdR-dependent manner (Bouillaut et al., 2013; Gu et al., 2017). We did not detect a SigL-dependent control of *cdsB* expression as previously observed (Gu et al., 2017) and only a two-fold decrease of expression was detected for three genes of the *prdA* operon in the *sigL::erm* mutant (Supplementary Table S7). For most of the genes very likely transcribed by SigL, the inducer is probably absent in TY medium and we were unable to detect a control by this sigma factor on their basal level of expression.

### Role of SigL in the Physiology of *C. difficile*

To complete our view of the role of SigL in the physiology of *C. difficile*, we combined the data on the control of expression by SigL with those obtained on the identification of “−24,−12” promoters. As described in other firmicutes (Deutscher et al., 2006; Francke et al., 2011; Nie et al., 2019), we observed SigL-dependent promoters upstream of 7 operons encoding phosphotransferase systems (PTS) belonging to the mannose (*CD0284*, *CD0490*, *CD3279*), cellobiose (*CD0860*) or mannitol/galactitol (*CD0040*, *CD2283*, *CD2327*) sub-families (Table 4 and Supplementary Figure S9). These PTS operons are associated with 5 LevR-type activators (CD0040, CD0283, CD2283, CD2328, CD3280) and a sixth one, a pseudogen, CD0858 (Stulke et al., 1998). Three operons encoding PTS (*CD0284*, *CD0490* and *CD2327*) were down-regulated in transcriptome (TY 4h of growth) or in qRT-PCR experiments (Table 4). These PTS are likely second-line systems used when glucose, fructose or mannitol are absent or depleted (Deutscher et al., 2006).

**FIGURE 7:**
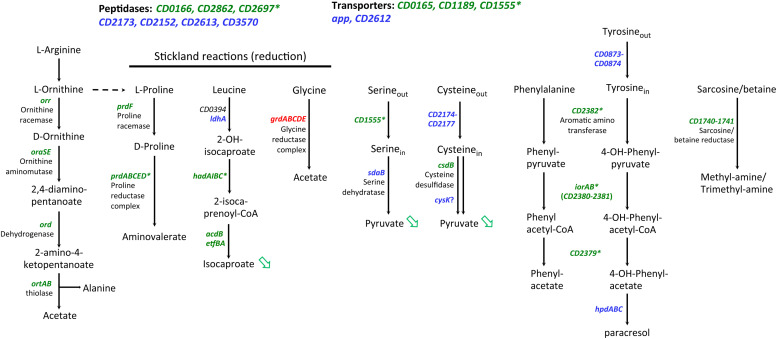
Genes involved in peptide and amino-acid catabolism controlled by SigL in *C. difficile.* Upstream of genes indicated in green, a “–24, –12” promoter was mapped or identified *in silico* (Table 4). * indicated genes with a “–24, –12” promoter and positively controlled by SigL in transcriptome or in qRT-PCR experiments (Table 4). Genes indicated in blue are positively controlled by SigL in transcriptome but a “–24, –12” promoter is absent upstream of the gene (Supplementary Table S7). Genes indicated in red are negatively controlled by SigL in transcriptome (Supplementary Table S7). Green arrow indicates a compound less detected by Gas-liquid chromatography analysis (Figure 8).

Interestingly, we also observed a large set of genes involved in amino-acid degradation or encoding peptidases and amino-acid permeases that are controlled either directly or indirectly by SigL and/or transcribed by the RNAP associated to SigL (Table 4 and Supplementary Table S7). *C. difficile* can use some amino acids as energy source through Stickland reactions (Neumann-Schaal et al., 2019). These reactions consist in the coupled fermentation of two amino acids in which one is oxidatively deaminated or decarboxylated and the other is reductively deaminated or reduced. The Stickland donors are valine, leucine, isoleucine and alanine while the acceptors are proline, glycine and leucine (Jackson et al., 2006). The *prdA* operon and the *prdC* gene, as well as the *hadA* operon required for the proline and the leucine reductive branch were transcribed by SigL and controlled by SigL-dependent EBPs, PrdR (Bouillaut et al., 2013) and probably LeuR (CD0402), respectively (Table 4 and Figure 7) (Nie et al., 2019). The expression of the *hadA* operon was strongly down-regulated in the *sigL::erm* mutant while the *prdA* operon was only weakly controlled by SigL in TY medium (Table 4). By contrast, the expression of the *grd* operon involved in glycine reduction was strongly up-regulated in the *sigL::erm* mutant compared to the wild-type strain (8 to 60-fold) (Supplementary Table S7). In *C. difficile*, SigL likely plays a key role in the hierarchy of amino acid utilization through Stickland reactions favoring the reductive degradation of proline and leucine as an energy source in detriment of glycine. A “−24,−12” promoter recognized by SigL was also mapped upstream of the *ord* operon involved in the oxidative degradation of ornithine (Fonknechten et al., 2009) associated with the EBP-OrdR. In addition, SigL seemed to control directly or indirectly the expression of genes involved in the uptake and degradation of aromatic amino acids (tyrosine and phenylalanine) (Bradshaw et al., 2019). SigL was also found to control the expression of genes involved in the degradation of serine (*sdaB*) and cysteine (*cdsB* and maybe also *cysK*) and encoding a probable serine/threonine exchanger (*CD1555*) or a transporter of cysteine/cystine (*CD2174* operon) (Dubois et al., 2016; Gu et al., 2017). Finally, a “−24,−12” promoter was mapped upstream of the *CD1740-CD1741* operon encoding a sarcosine reductase associated with a two-component system, SarR-SarS. In conclusion, SigL plays a crucial role in the control of peptide and amino acid catabolism.

### Impact of SigL Inactivation on Growth and End-Fermentation Products

**FIGURE 8:**
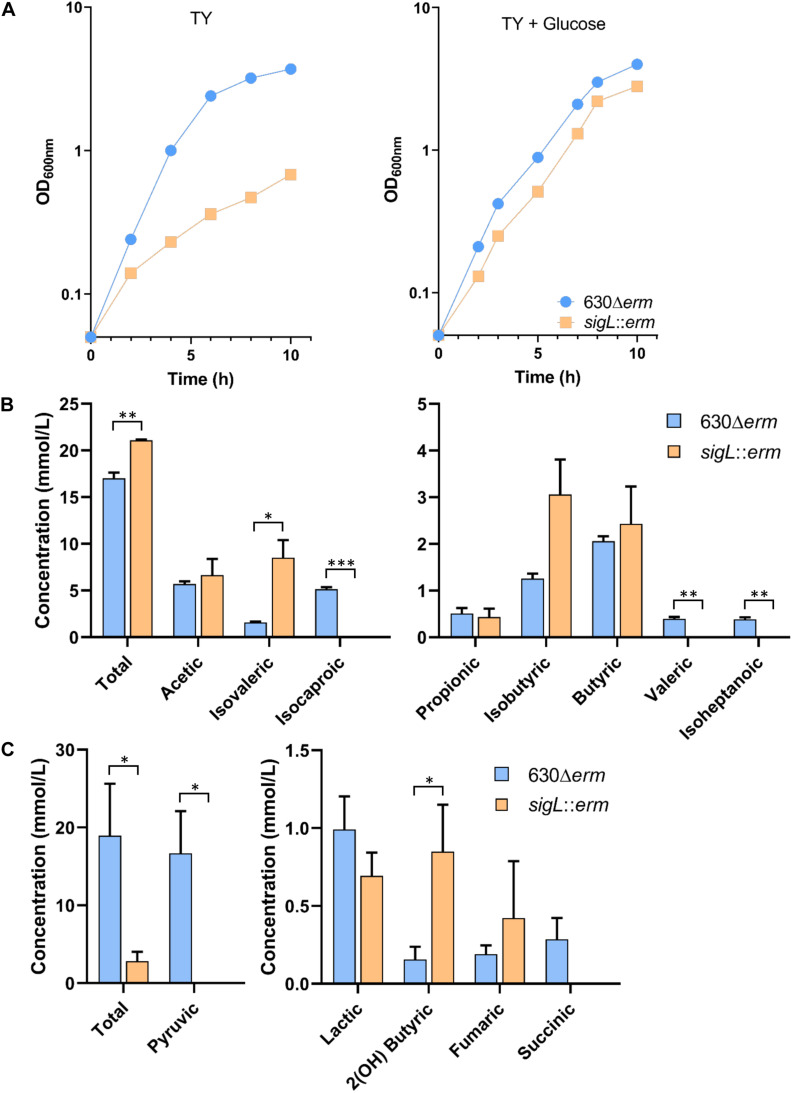
Growth and fermentation products of the WT and *sigL::erm* mutant strains. **(A)** Growth curves of the 630Δ*erm* and the *sigL*::*erm* strains in TY and TY + 0.5% glucose. Growth curves are representative of at least three independent experiments. Concentrations of volatile **(B)** and non-volatile **(C)** fermentations end products of the 630Δ*erm* and the *sigL*::*erm* strains grown 48 h in TY. Gas-liquid chromatography analysis was performed from the supernatant of the culture of both strains as described in materials and methods. The concentration of the fermentation end products was standardized on the OD_600nm_ of the cultures after 48 h of growth. We could not detect butanol nor ethanol by these assays. The error bars represent the standard deviation of the mean. Asterisks indicate statistical significance (*t*-test or *t*-test Welch, ^∗∗∗^*p* ≤ 0.001, ^∗∗^*p* ≤ 0.01, ^∗^*p* ≤ 0.05).

To confirm the role of SigL in *C. difficile*, we tested the growth of the WT and *sigL*::*erm* mutant strains (Dubois et al., 2016) in TY medium (Figure 8A). The mutant inactivated for SigL showed a reduced growth rate as compared to the strain 630Δ*erm* (Figure 8A). This result is in agreement with the proposed key role of SigL in the degradation of amino acids that are probably used as energy sources by *C. difficile* in TY. We then tested the effect on growth of the addition of glucose or glycine since the *grd* operon encoding the glycine reductase is not transcribed from a “−24,−12” promoter. Interestingly, the addition of glucose to TY partially restored the growth of the *sigL::erm* mutant (Figure 8A) but this was not the case for the addition of glycine (data not shown). These results suggested that the addition of glucose rerouting the metabolism (Antunes et al., 2012) allows to compensate the reduced utilization of peptides while glycine is not sufficient to restore growth even if the *grd* operon is strongly up-regulated (8- to 60-fold) in the *sigL::erm* mutant (Supplementary Table S7).

To confirm the impact of SigL inactivation on the fermentation processes, we also analyzed the end products of fermentation in the 630Δ*erm* and of the *sigL::erm* mutant by gas-liquid chromatography after 48 h of growth in TY medium. While the total concentration of volatile acids slightly increased, we observed a drastic decrease of non-volatile acids in the *sigL::erm* mutant compared to the 630Δ*erm* strain (Figures 8B,C). These results confirmed that SigL inactivation led to important changes in metabolism and metabolite production. We observed a drastic depletion of pyruvic acid (Figure 8C) that can be associated at least partly to a drop-in expression of the genes involved in cysteine and serine uptake and degradation (Figure 7) and to an increased expression of *ldh* and maybe also *adhE* (Supplementary Figure S9). In the *sigL* mutant, the strong decreased production of the end-product of leucine degradation, isocaproic acid, (Figure 8B) correlates with the drastic reduction of expression of the *hadA* operon (Figure 7). Decreased leucine degradation through the reductive Stickland reactions could redirect leucine catabolism to the oxidative Stickland reactions explaining the significant increase of isovaleric acid in the supernatant of the *sigL::erm* mutant (Figure 8B) (Neumann-Schaal et al., 2015). The apparent depletion of succinate might result from the strong up-regulation of the pathway of conversion of succinate to crotonyl-CoA (Supplementary Figure S9). Globally, the effects observed on metabolites could also result from a change in the metabolism balance because of the lack of SigL, to an indirect influence of SigL on the fermentation pathways through the modulation of intracellular concentration of metabolites or a cascade of regulation by uncharacterized mechanisms. It is worth noting that a large set of transcriptional regulators are up- or down-regulated in the *sigL::erm* mutant (Supplementary Table S7) allowing indirect metabolic controls.

## Conclusion

In the present study, we provide the first transcriptional map of the *C. difficile* genome demonstrating a complex structure of transcriptional units and operon organization in this pathogen. We have applied the combination of *in silico* and experimental strategies to establish the list of proposed TSS that will serve as a start point for further studies in global and gene-specific scale. In addition to primary TSSs, this genome-wide TSS mapping revealed the presence of tandem and internal promoters suggesting alternative ways to accommodate gene expression changes during *C. difficile* development. This pathogen uses its large arsenal of sigma factors to determine the promoter selectivity. *C. difficile* has also two housekeeping SigA encoding genes, one transcribed from a SigH-dependent promoter (Saujet et al., 2011) and the second one likely in operon with *dnaG* and an unexpected number of extended −10 boxes. This work extends our knowledge on the expression program during stationary phase (mediated by stationary phase sigma factors, SigH and SigB) and sporulation. The promiscuity for the sequence recognition for the SigF and SigG sigma factors suggests the existence of a less controlled forespore program compared to *B. subtilis* as previously proposed (Saujet et al., 2013, 2014; Fimlaid et al., 2013). Finally, we identify several regulators likely expressed under the control of sporulation specific sigma factors (CD0629 and CD2316 by SigE, CD2599 by SigF or SigG) in addition to conserved SpoIIID and SpoVT transcriptional regulators (Saujet et al., 2014) suggesting that additional uncharacterized mechanisms of sporulation regulation might exist in *C. difficile*. Focusing on one of important alternative sigma factors, we characterized here the SigL regulon largely involved in amino-acid degradation pathways as a key trait for the colonization of the gut. In addition to determination of TSS, valuable information could be deduced from 5′-end RNA-seq on the potential RNA processing events impacting the RNA stability, as well as on the 5′ untranslated regions harboring important regulatory motifs for transcriptional and post-transcriptional regulatory mechanisms. These data complete out previous genome-wide identification of ncRNAs in *C. difficile* linking together different facets of global regulatory networks in this pathogen. This study thus constitutes the first essential step towards better understanding of the complex transcriptional and post-transcriptional regulations governing the infection cycle and adaptation of this successful enteropathogen to host environments.

## Data Availability Statement

The datasets presented in this study can be found in online repositories. The names of the repository/repositories and accession number(s) can be found in the article/ Supplementary Material.

## Author Contributions

OS and IM-V co-designed the study, analyzed the data and wrote the manuscript. OS, TD, and LS performed the experiments. MM, PS, and MG collected and analyzed the data *in silico*. OS, IM-V, LS, PB, and BD manually analyzed the TSS. TD collected and analyzed the transcriptomic data. BD supervised the experimental work. TD, MM, MG, and BD revised the manuscript. All authors contributed to the article and approved the submitted version.

## Conflict of Interest

The authors declare that the research was conducted in the absence of any commercial or financial relationships that could be construed as a potential conflict of interest.

## Footnotes

1 www.bioconductor.org

## Supplementary Material

FIGURE S1 Representative example of TSS identification by PhageTerm software. Cov2HTML (Monot et al., 2014) visualization of 5′-end RNA-seq data for TAP−/TAP+ profile comparison is presented in the left panel and PhageTerm (Garneau et al., 2017). TSS identification is shown in the right panel.

FIGURE S2 Operon organization of the genes coding the proteins involved in flagella biosynthesis and function associated with TSS mapping. The flagella genes are organized in several transcriptional units driven by RNAP in complex with alternative flagellum SigD or housekeeping SigA sigma factors. The TSS are shown by broken arrows, SigD-associated promoters are indicated in blue, SigA-associated promoters are shown in green and undefined promoters are depicted in black. Three boxed images show a zoom to the TSS mapping visualization for selected promoters. The genomic region visualization was made with MAGE platform (Vallenet et al., 2020), coding sequences are indicated by ORF in red and previously identified noncoding RNAs are shown by blue-green boxes.

FIGURE S3 Nucleotide composition of TSS and frequency of each initiation nucleotide in *C. difficile.* 100 nucleotide regions (from −99 to +1) upstream of the TSS for *C. difficile* strain 630 genes have been aligned and the deduced consensus sequence generated with Weblogo program is presented. “+1” indicates TSS position, “−10” indicates the position of −10 promoter element.

FIGURE S4 Length distribution of 5′UTR regions in the *C. difficile* strain 630 genome. The graph shows the length of 5′UTR (distance from the TSS to the translational start) and the number of genes with corresponding 5′UTR length range.

FIGURE S5 TSS mapping of dual **(A)** and internal **(B)** promoters. Representative examples of 5′-end RNA-seq (TAP−/TAP+ profile comparison) and RNA-seq data for dual tandem TSS and internal TSS inside the coding sequences are shown in panels **(A,B)**, respectively. Cov2HTML (Monot et al., 2014) was used for the visualization. On a RNA-seq and 5′-end RNA-seq sequence read mapping visualization, coding sequences are indicated by blue arrows. The 5′-end RNA-seq data for either positive “strand +” or negative “strand −” strands are presented in the panels. The TSS identified by 5′-end RNA-seq are indicated by red broken arrows and potential processing sites are indicated by scissors mark. Sigma factor consensus associated with a given TSS is indicated. The TSS corresponds to a position with significantly greater number of reads in TAP+ sample, potential cleavage site corresponds to position with large number of reads in both TAP- and TAP+ samples. 5′-end RNA-seq data show 51-bp reads matching to the 5′-transcript ends, while RNA-seq data show reads covering whole transcript.

FIGURE S6 Examples of cleavage sites detected by TSS-mapping. Representative examples of 5′-end RNA-seq (TAP−/TAP+ profile comparison) and RNA-seq data for complex potential processing profiles are shown. The RNA-seq and 5′-end RNA-seq data visualization is presented as in Figure 1. Potential cleavage site shown by scissors mark corresponds to a position with large number of reads in both TAP− and TAP+ samples. RBS are shown by green boxes to highlight the positions of potential cleavage sites in the proximity or inside RBS.

FIGURE S7 Additional examples of promoters controlling regulatory genes detected by TSS-mapping. Representative examples of 5′-end RNA-seq (TAP−/TAP+ profile comparison) data for the identification of TSS for genes encoding important transcriptional regulators are shown. The 5′-end RNA-seq data visualization is presented as in Figure 1. The sequence of promoter region is shown upstream of TSS with the −35 and −10 promoter elements indicated in blue and TSS indicated in red.

FIGURE S8 Confusion matrix on training with the standard and strict criterion. Confusion matrices for the training set. **(A)** Listing scheme as in Figure 4, **(B)** Strict listing scheme with one top-scoring sigma factor taken as the prediction.

FIGURE S9 Carbon metabolism genes controlled by SigL in *C. difficile*. Upstream of genes indicated in green, a “−24, −12” promoter was mapped or identified *in silico* (Table 4). ^∗^ indicated genes with a “−24, −12” promoter (Table 4). Genes in green are positively controlled by SigL in transcriptome or in qRT-PCR experiments as indicated by #. Genes indicated in red are negatively controlled by SigL in transcriptome (Supplementary Table S7). Green arrow indicates a compound less detected by Gas-liquid chromatography analysis (Figure 8).

TABLE S1 Strains and plasmids used in this study.

TABLE S2 Complete list of TSS and predicted promoters for *C. difficile* 630 CDS genes.

TABLE S3 Complete list of TSS and predicted promoters for *C. difficile* 630 ncRNA genes.

TABLE S4 *In silico* association of TSS with known sigma factors in *C. difficile*.

TABLE S5 Identification of promoters recognized by SigF and SigG.

TABLE S6 Identification of promoters recognized by SigE and/or SigK.

TABLE S7 Transcriptomics results for *sigL::erm* mutant.
