# Supplementary material for: Genome-Wide Transcription Start Site Mapping and Promoter Assignments to a Sigma Factor in the Human Enteropathogen Clostridioides difficile
Source: Front Microbiol. 2020 Aug 13;11:1939. doi: 10.3389/fmicb.2020.01939 (PMC7438776; doi:10.3389/fmicb.2020.01939)

Figure S1.

TSS-PhageTerm = TSS-Manuel (74%)

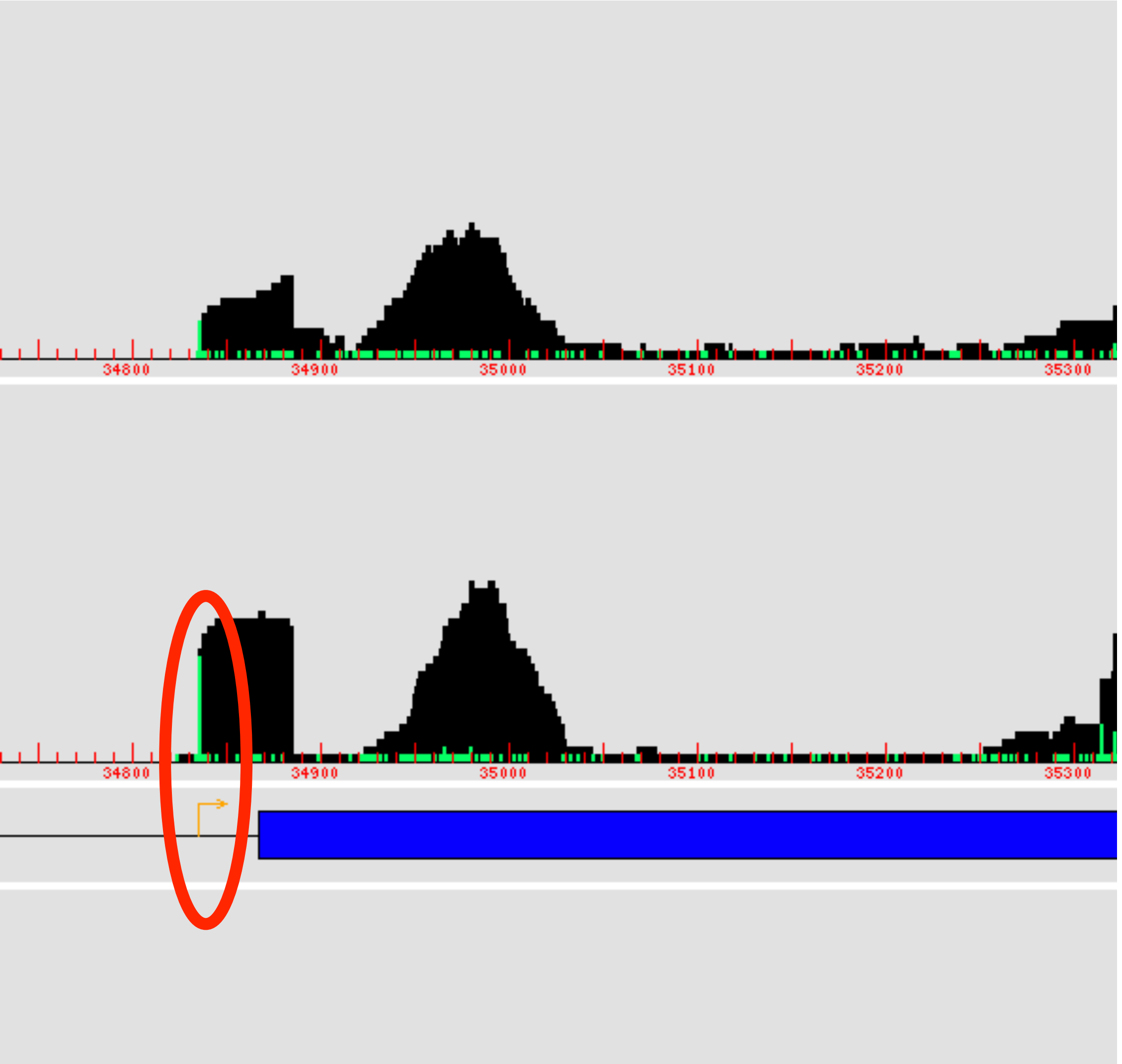

CD630_00210_tap PhageTerm Analysis

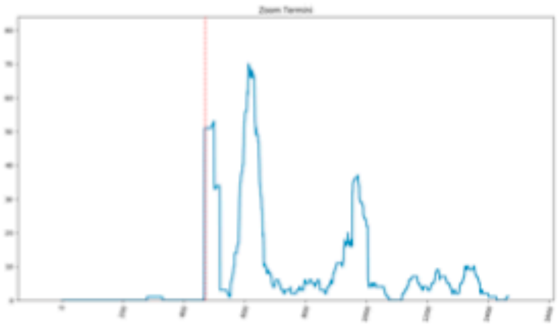

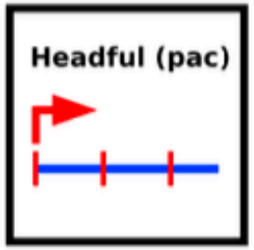

PhageTerm Method

Ends	Left (red)	Right (green)	Permuted	Orientation	Class	Type
Redundant	469	Distributed	Yes	Forward	Headful (pac)	P1

Strand	Location	T	pvalue
+	469	0.98	2.54e-11
	3532	0.69	2.89e-22
	955	0.50	1.51e-06
	1324	0.30	1.00e+00
	568	0.29	1.00e+00
-	1	0.00	1.00e+00
	2613	0.00	1.00e+00
	2615	0.00	1.00e+00
	2616	0.00	1.00e+00
	2617	0.00	1.00e+00

T (Start. Pos. Cov. / Whole Cov.)

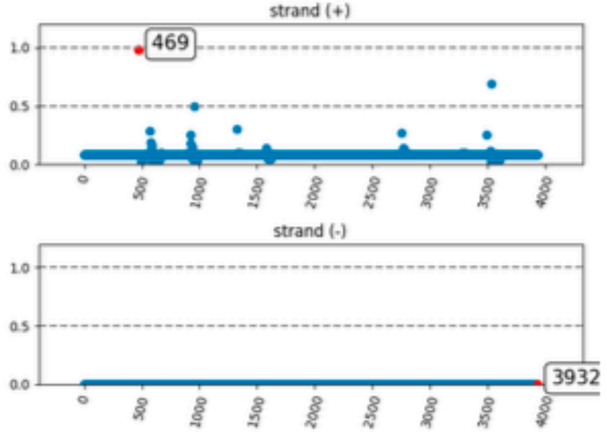

Supplement: FIGURE S1 — Representative example of TSS identification by PhageTerm software. Cov2HTML (Monot et al., 2014) visualization of 5′-end RNA-seq data for TAP−/TAP+ profile comparison is presented in the left panel and PhageTerm (Garneau et al., 2017). TSS identification is shown in the right panel. [file Data_Sheet_1.PDF]
