# Supplementary figures and images for: Genome-Wide Transcription Start Site Mapping and Promoter Assignments to a Sigma Factor in the Human Enteropathogen Clostridioides difficile
Source: Front Microbiol. 2020 Aug 13;11:1939. doi: 10.3389/fmicb.2020.01939 (PMC7438776; doi:10.3389/fmicb.2020.01939)

Figure S2.

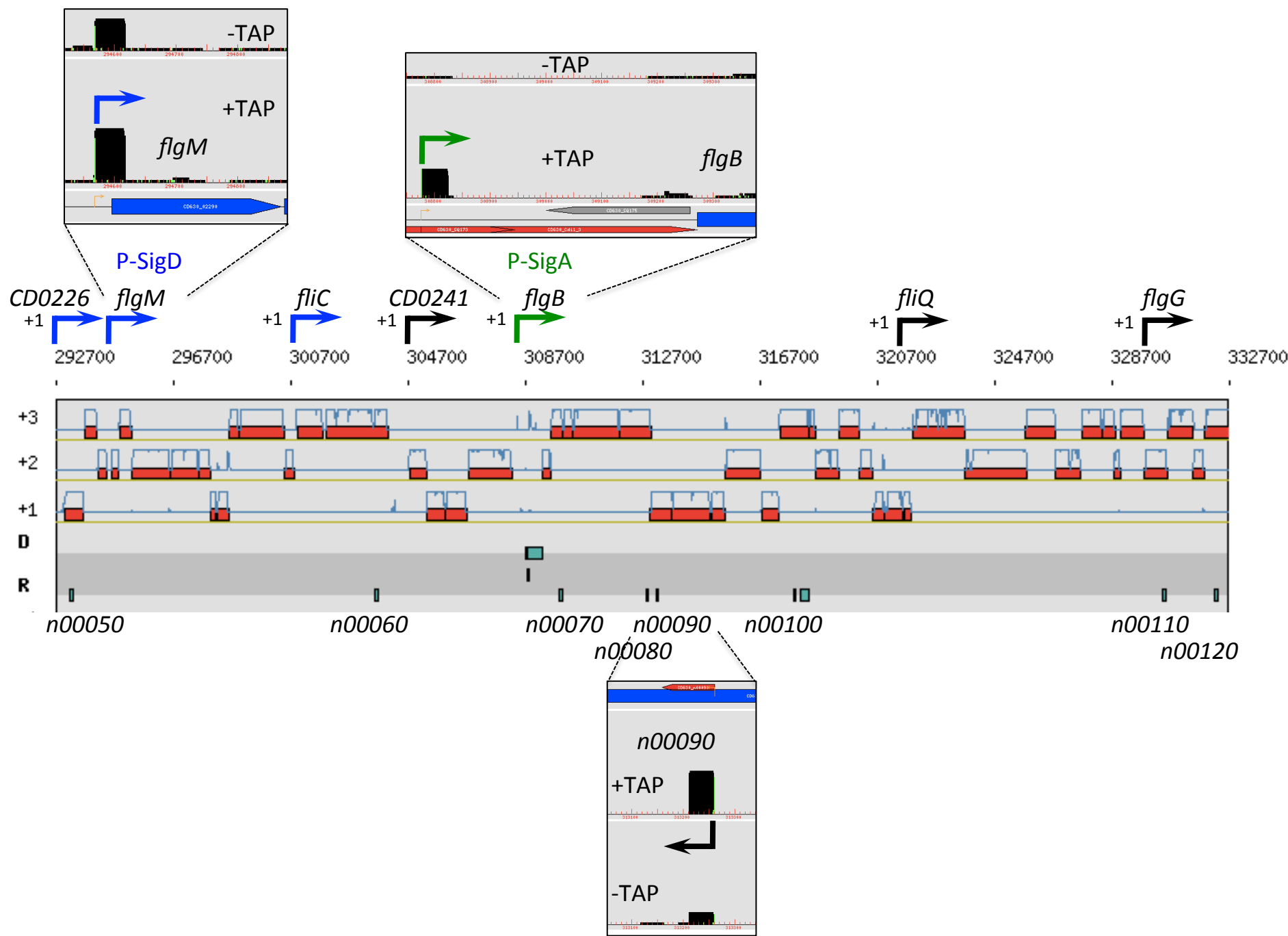

Supplement: FIGURE S2 — Operon organization of the genes coding the proteins involved in flagella biosynthesis and function associated with TSS mapping. The flagella genes are organized in several transcriptional units driven by RNAP in complex with alternative flagellum SigD or housekeeping SigA sigma factors. The TSS are shown by broken arrows, SigD-associated promoters are indicated in blue, SigA-associated promoters are shown in green and undefined promoters are depicted in black. Three boxed images show a zoom to the TSS mapping visualization for selected promoters. The genomic region visualization was made with MAGE platform (Vallenet et al., 2020), coding sequences are indicated by ORF in red and previously identified noncoding RNAs are shown by blue-green boxes. [file Data_Sheet_2.PDF]

Figure S3.

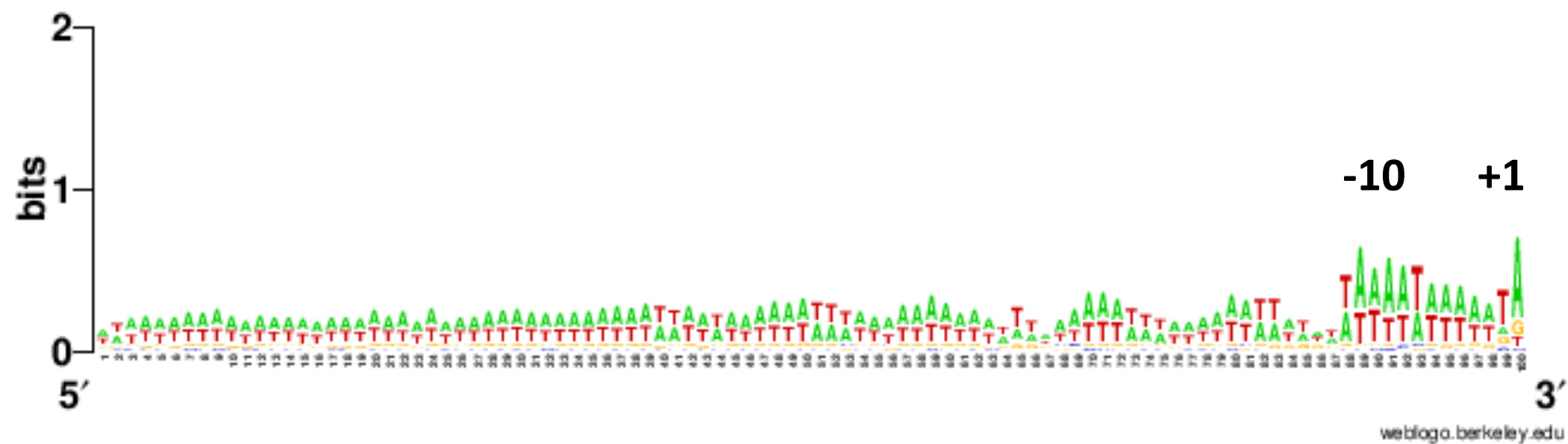

Supplement: FIGURE S3 — Nucleotide composition of TSS and frequency of each initiation nucleotide in C. difficile. 100 nucleotide regions (from −99 to +1) upstream of the TSS for C. difficile strain 630 genes have been aligned and the deduced consensus sequence generated with Weblogo program is presented. “+1” indicates TSS position, “−10” indicates the position of −10 promoter element. [file Data_Sheet_3.PDF]

Figure S4.

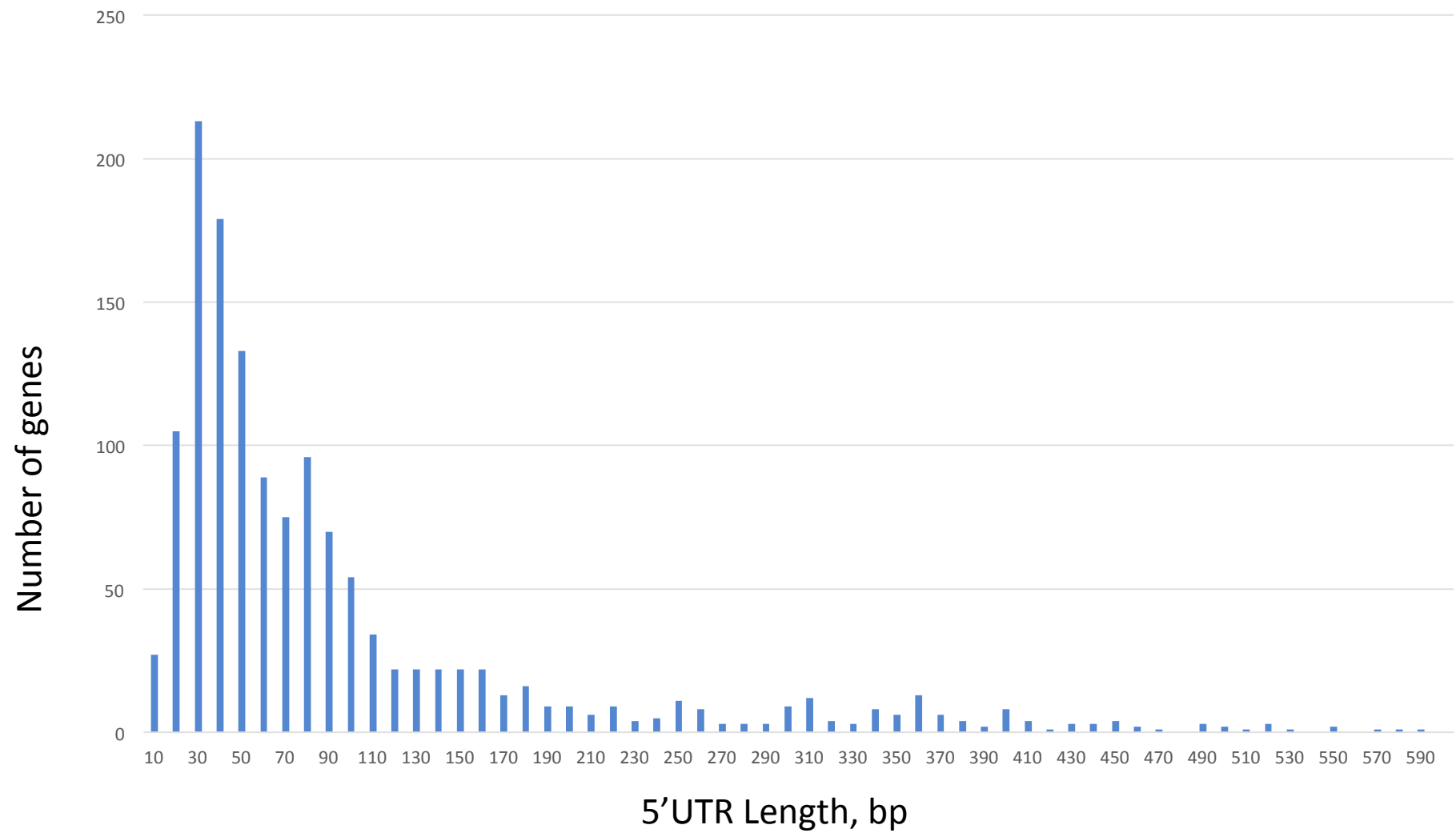

Supplement: FIGURE S4 — Length distribution of 5′UTR regions in the C. difficile strain 630 genome. The graph shows the length of 5′UTR (distance from the TSS to the translational start) and the number of genes with corresponding 5′UTR length range. [file Data_Sheet_4.PDF]

Figure S5.

A

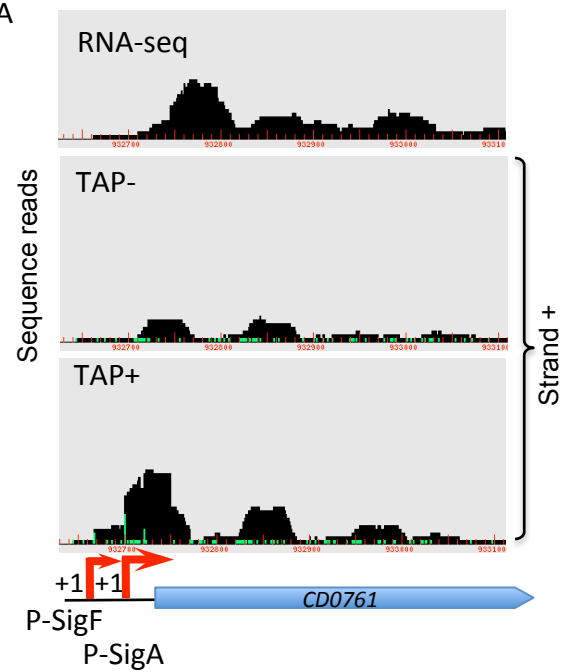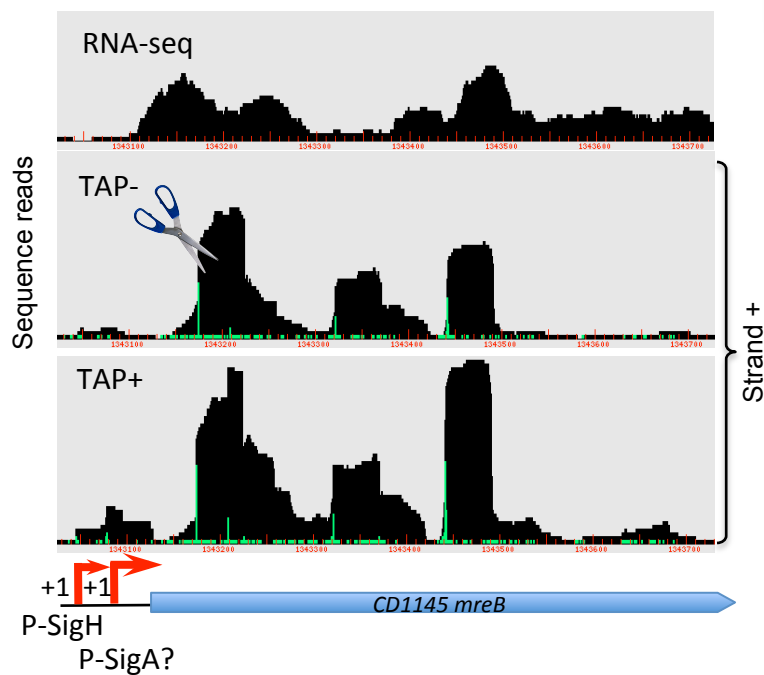

B

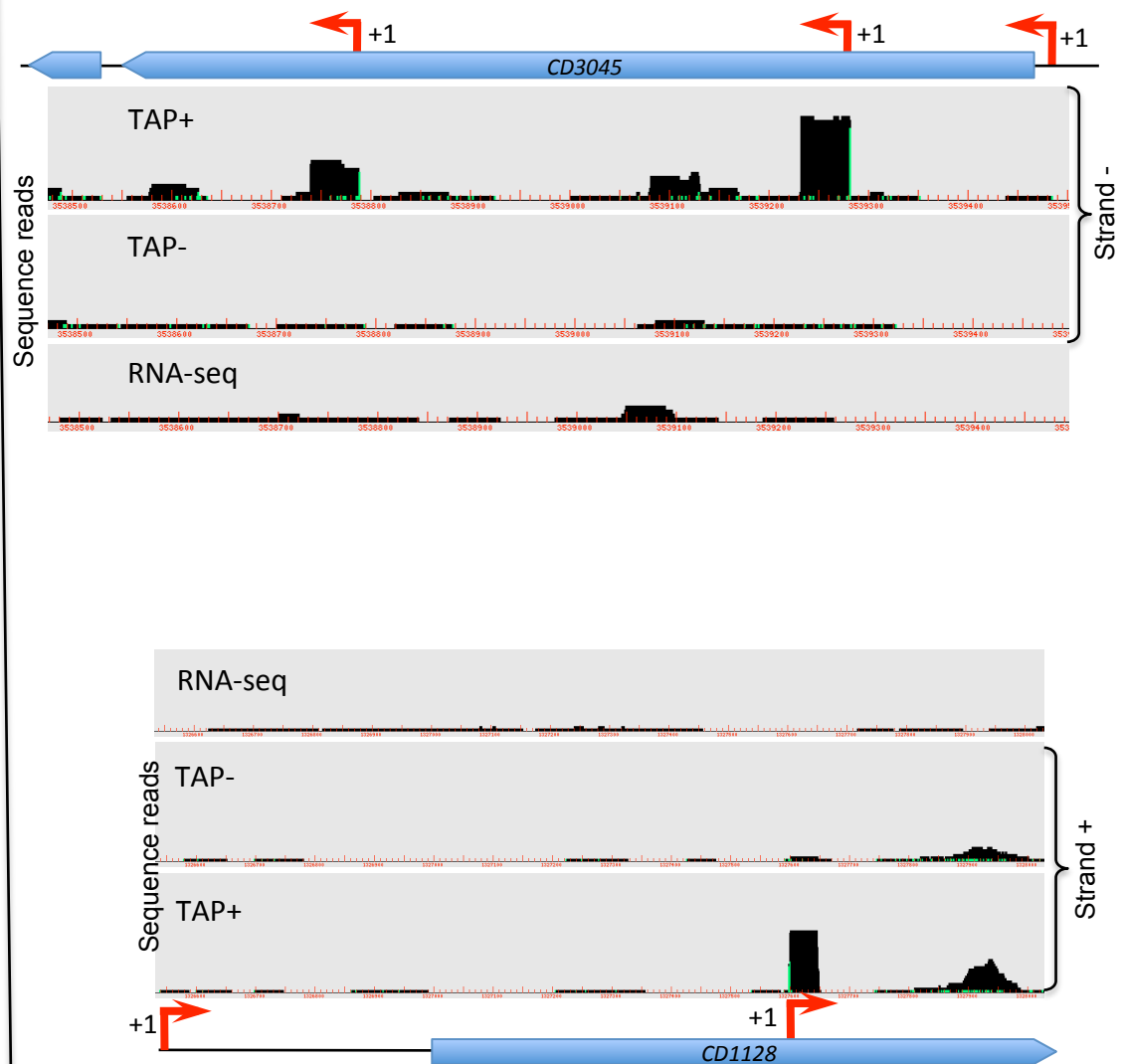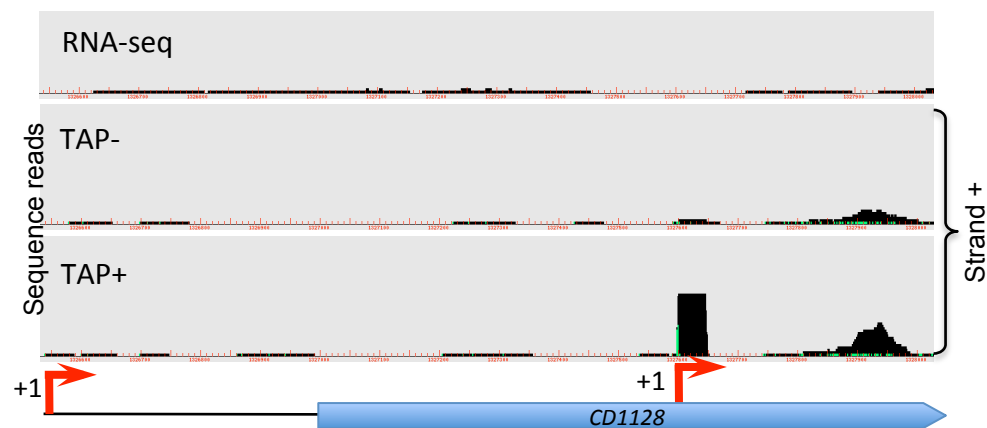

Supplement: FIGURE S5 — TSS mapping of dual (A) and internal (B) promoters. Representative examples of 5′-end RNA-seq (TAP−/TAP+ profile comparison) and RNA-seq data for dual tandem TSS and internal TSS inside the coding sequences are shown in panels (A,B), respectively. Cov2HTML (Monot et al., 2014) was used for the visualization. On a RNA-seq and 5′-end RNA-seq sequence read mapping visualization, coding sequences are indicated by blue arrows. The 5′-end RNA-seq data for either positive “strand +” or negative “strand −” strands are presented in the panels. The TSS identified by 5′-end RNA-seq are indicated by red broken arrows and potential processing sites are indicated by scissors mark. Sigma factor consensus associated with a given TSS is indicated. The TSS corresponds to a position with significantly greater number of reads in TAP+ sample, potential cleavage site corresponds to position with large number of reads in both TAP- and TAP+ samples. 5′-end RNA-seq data show 51-bp reads matching to the 5′-transcript ends, while RNA-seq data show reads covering whole transcript. [file Data_Sheet_5.PDF]

Figure S6.

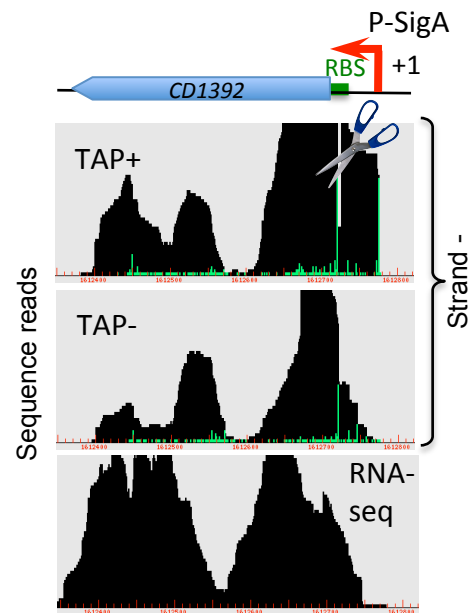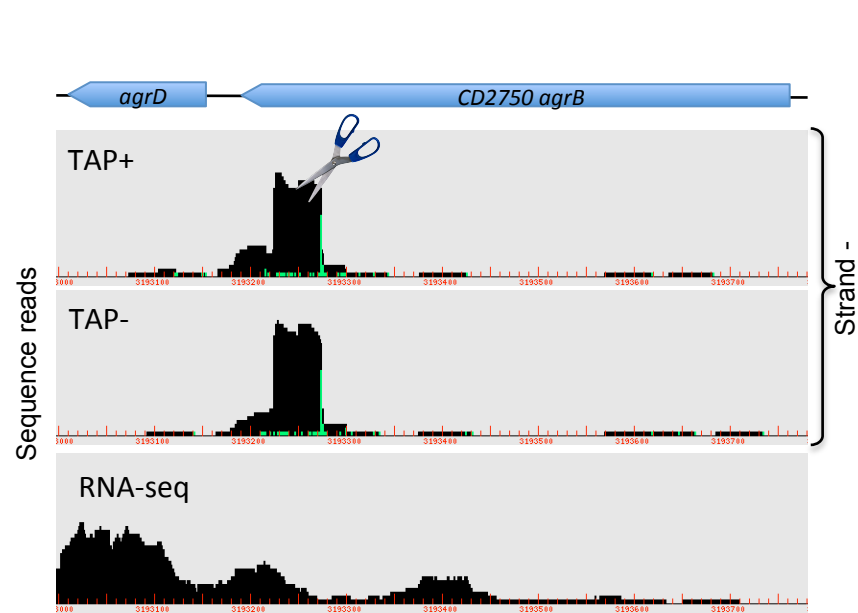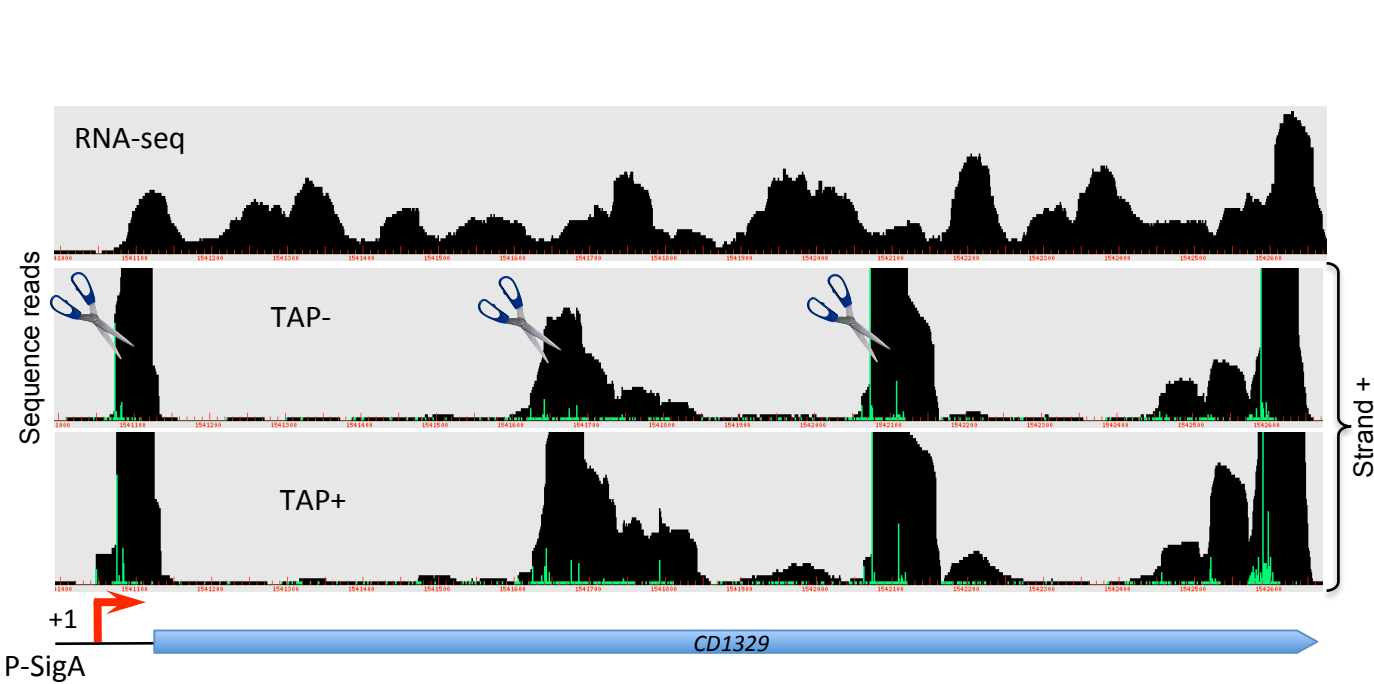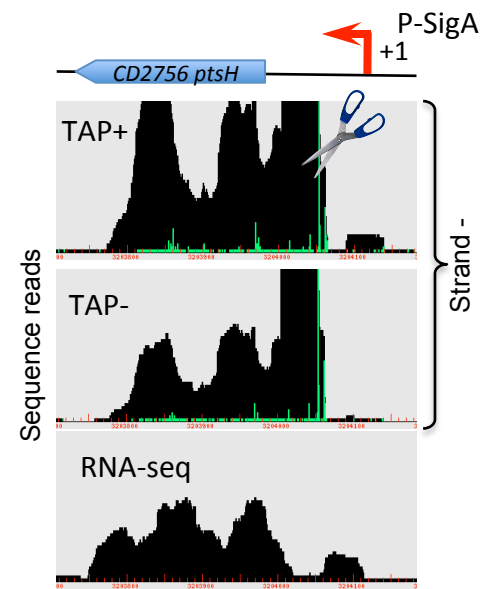

Supplement: FIGURE S6 — Examples of cleavage sites detected by TSS-mapping. Representative examples of 5′-end RNA-seq (TAP−/TAP+ profile comparison) and RNA-seq data for complex potential processing profiles are shown. The RNA-seq and 5′-end RNA-seq data visualization is presented as in Figure 1. Potential cleavage site shown by scissors mark corresponds to a position with large number of reads in both TAP− and TAP+ samples. RBS are shown by green boxes to highlight the positions of potential cleavage sites in the proximity or inside RBS. [file Data_Sheet_6.PDF]

Figure S8.

A

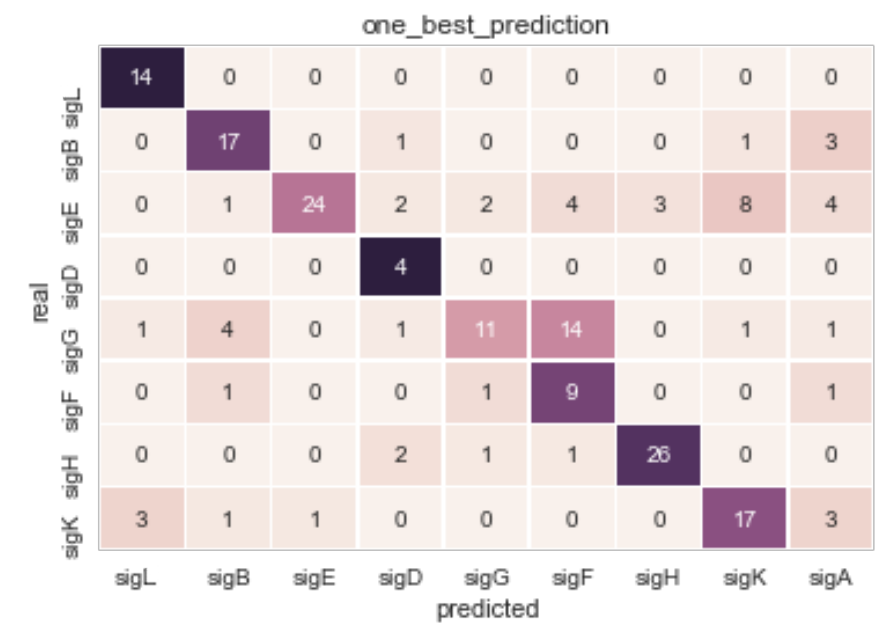

B

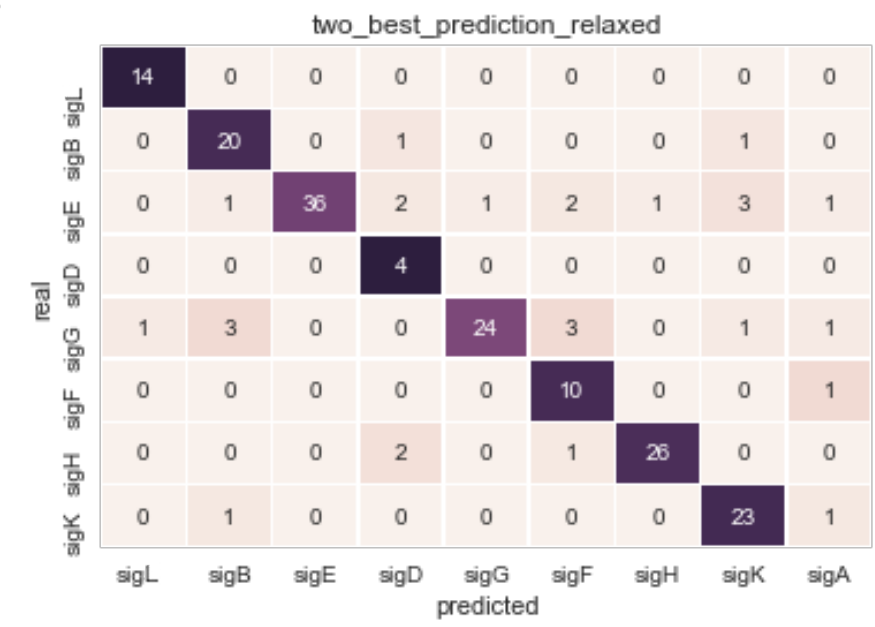

Supplement: FIGURE S8 — Confusion matrix on training with the standard and strict criterion. Confusion matrices for the training set. (A) Listing scheme as in Figure 4, (B) Strict listing scheme with one top-scoring sigma factor taken as the prediction. [file Data_Sheet_8.PDF]

Figure S9.

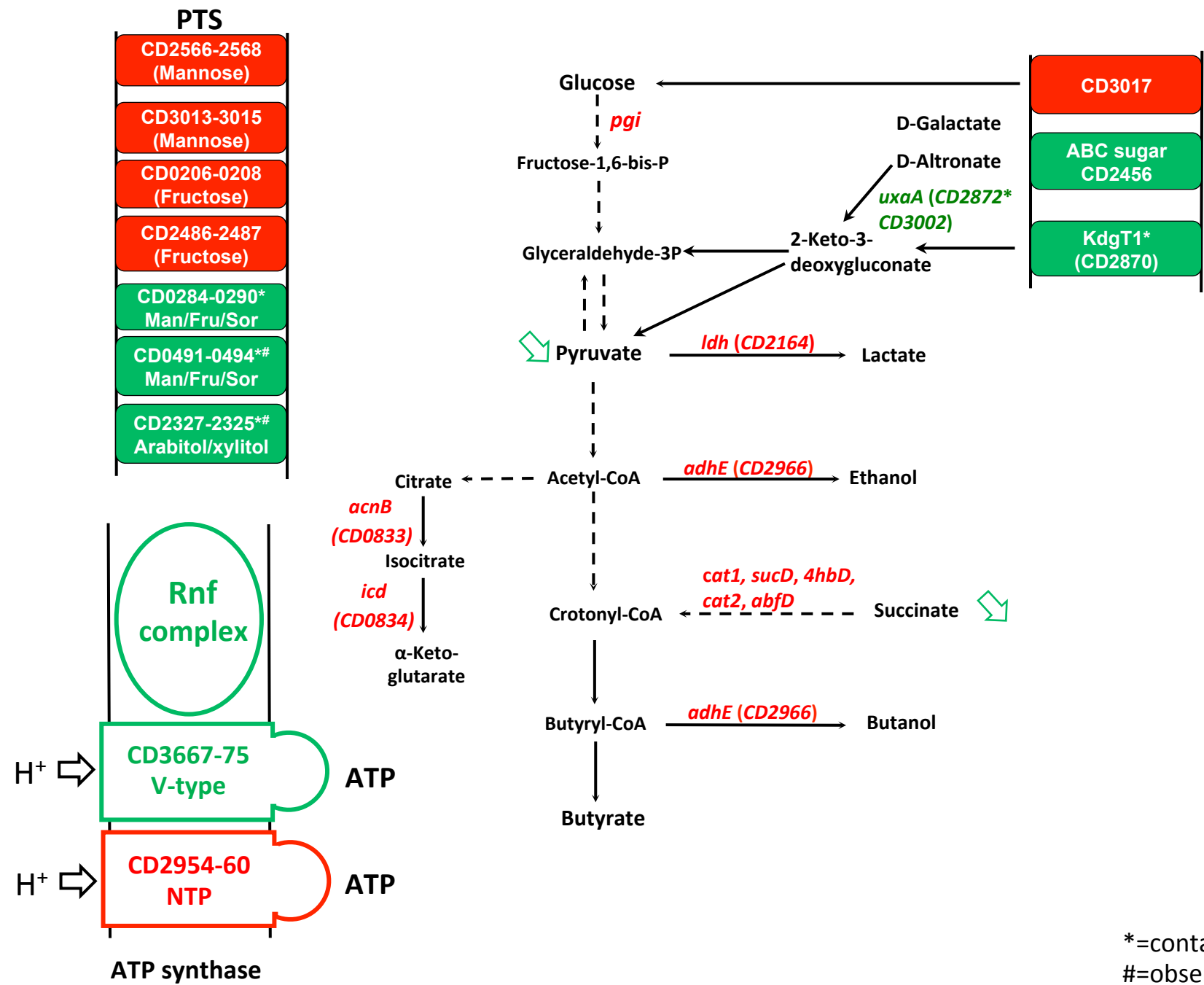

Supplement: FIGURE S9 — Carbon metabolism genes controlled by SigL in C. difficile. Upstream of genes indicated in green, a “−24, −12” promoter was mapped or identified in silico (Table 4). ∗ indicated genes with a “−24, −12” promoter (Table 4). Genes in green are positively controlled by SigL in transcriptome or in qRT-PCR experiments as indicated by #. Genes indicated in red are negatively controlled by SigL in transcriptome (Supplementary Table S7). Green arrow indicates a compound less detected by Gas-liquid chromatography analysis (Figure 8). [file Data_Sheet_9.PDF]
