# Supplementary material for: Genome-Wide Transcription Start Site Mapping and Promoter Assignments to a Sigma Factor in the Human Enteropathogen Clostridioides difficile
Source: Front Microbiol. 2020 Aug 13;11:1939. doi: 10.3389/fmicb.2020.01939 (PMC7438776; doi:10.3389/fmicb.2020.01939)
Supplement: FIGURE S7 — Additional examples of promoters controlling regulatory genes detected by TSS-mapping. Representative examples of 5′-end RNA-seq (TAP−/TAP+ profile comparison) data for the identification of TSS for genes encoding important transcriptional regulators are shown. The 5′-end RNA-seq data visualization is presented as in Figure 1. The sequence of promoter region is shown upstream of TSS with the −35 and −10 promoter elements indicated in blue and TSS indicated in red. [file Data_Sheet_7.PDF]

Figure S7.

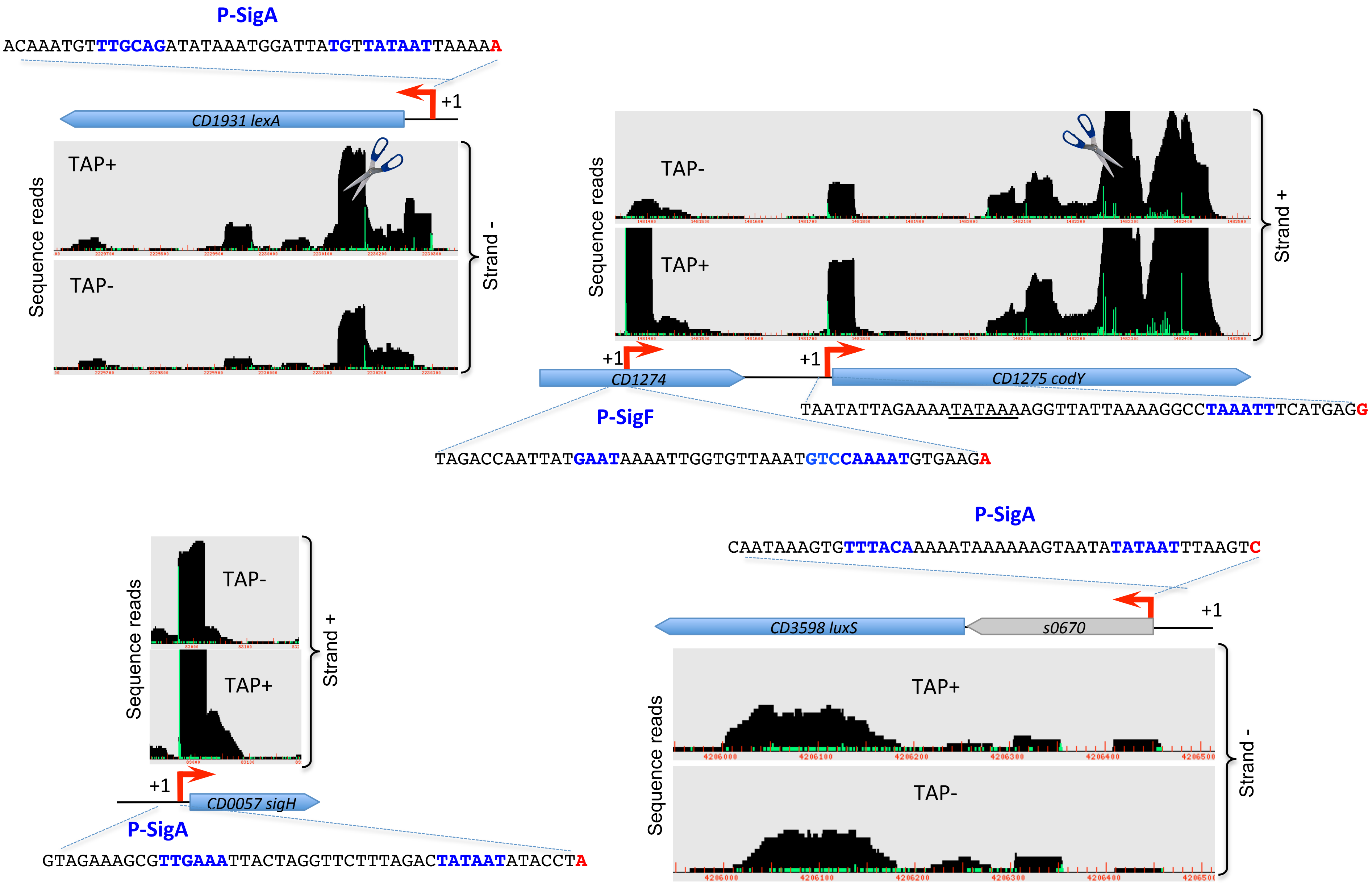

P-SigA
ACAAATGTTTGCAGATATAAATGGATTATGTTATAATTAAAAA
+1
CD1931 lexA
TAP+
TAP-
Sequence reads
Strand -
TAP-
TAP+
Sequence reads
Strand +
+1
+1
CD1274
CD1275 codY
TAATATTAGAAAATATAAAAGGTTATTAAAAGGCCTAAATTTCATGAGG
P-SigF
TAGACCAATTATGAATAAAATTGGTGTTAAATGTCCAAAATGTGAAGA
TAP-
TAP+
Sequence reads
Strand +
+1
CD0057 sigH
P-SigA
GTAGAAAGCGTTGAAATTACTAGGTTCTTTAGACTATAATATACCTA
P-SigA
CAATAAAGTGTTTACAAAAATAAAAAAGTAATATATAATTTAAGTC
+1
CD3598 luxS
s0670
TAP+
TAP-
Sequence reads
Strand -
