# Supplementary material for: Genome-Wide Transcription Start Site Mapping and Promoter Assignments to a Sigma Factor in the Human Enteropathogen Clostridioides difficile
Source: Front Microbiol. 2020 Aug 13;11:1939. doi: 10.3389/fmicb.2020.01939 (PMC7438776; doi:10.3389/fmicb.2020.01939)
Supplement: TABLE S1 — Strains and plasmids used in this study. [file Table_1.pdf]

Table S1. Strains and plasmids used in this study

Strains	Genotype	Origin
E. coli		
TOP 10	F^- *mcr*A D(*mrr-hsd*RMS-*mcr*BC) f80*lac*ZDM15 D*lac*X74 *deo*R, *rec*A1 *ara*D139 D(*ara-leu*)7697 *gal*K *rps*L(StrR) *end*A1 *nup*G	Invitrogen
HB101 (RP4)	*supE*44 *aa*14 *galK*2 *lacY*1 Δ(*gpt-proA*) 62 *rpsL*20 (Str^R)*xyl-5 mtl-1 recA*13 Δ(*mcrC-mrr*) *hsdS*$_B$($r_B^- m_B^-$) RP4 (Tra^+ IncP Ap^R Km^R Tc^R)	laboratory stock
C. difficile		
630 Δ*erm*	630Δ*erm*	laboratory stock
CDIP217	630Δ*erm sigL*::*erm*	Dubois et al, 2016
CDIP342	630Δ*erm sigL*::*erm* pMTL84121-*sigL*	Dubois et al, 2016
